# Lipid Signaling Modulates the Response to Fumonisin Contamination and Its Source, *Fusarium verticillioides*, in Maize

**DOI:** 10.3389/fpls.2021.701680

**Published:** 2021-11-08

**Authors:** Laura Righetti, Chiara Dall’Asta, Luigi Lucini, Paola Battilani

**Affiliations:** ^1^Department of Food and Drug, University of Parma, Parma, Italy; ^2^Department for Sustainable Food Process, Università Cattolica del Sacro Cuore, Piacenza, Italy; ^3^Department of Sustainable Crop Production, Università Cattolica del Sacro Cuore, Piacenza, Italy

**Keywords:** fumonisins, metabolomics, lipidomics, oxylipins, mycotoxins

## Abstract

Fumonisin-contaminated maize (*Zea mays* L.) products are a major health concern because of their toxic effects in humans and animals. Breeding maize for increased mycotoxin resistance is one of the key sustainable strategies for mitigating the effects of fumonisin contamination. Recent studies suggest a link between fumonisin accumulation and plant lipid and oxylipin profiles. However, the data collected so far do not reveal a cause-and-effect relationship. In this study, to decipher the multifactorial nature of mycotoxin resistance and plant–pathogen interaction mechanisms, we examined the oxylipin and complex lipid profiles of two maize hybrids (H21 and H22, the latter showing significantly lower FBs content) grown in the open field in two locations over 3years. Untargeted ultra-high performance liquid chromatography coupled with quadrupole-time-of-flight (UHPLC-Q-TOF), together with chemometrics analysis, successfully distinguished between the two hybrids as having low- and high-level fumonisin contamination. Considering that H21 and H22 were exposed to the same environmental factors, the higher activation of lipid signaling systems in H22 suggests that other routes are enabled in the less susceptible hybrids to limit fumonisin B (FB) accumulation. Our results highlighted the crucial role played by oxylipin and sphingolipid signaling in modulating the complex maize response to *F. verticillioides* infection. Overall, our results returned a global view on the changes in lipid metabolites related to fumonisin accumulation under open field conditions, and revealed a strong activation of the lipid signaling cascade in maize in the presence of FB_1_.

## Introduction

Climate change has been recognized as the major driver of the increase in mycotoxin occurrence/co-occurrence in food and feed commodities worldwide (Battilani et al., 2016; Leggieri et al., 2020). Additionally, the current environmental conditions have altered the prevalence of pathogens as well as their interactions with diverse plant species in the field, leading to quantitative and qualitative yield losses and the accumulation of mycotoxins in crop plants.

*Fusarium verticillioides*, the main member of the *Gibberella fujikuroi* species complex (GFSC), is a fungus that causes ear rot in maize (*Zea mays* L.) in the field, and produces a wide spectrum of fumonisins, among which fumonisin B_1_ (FB_1_), FB_2_, and FB_3_ are the most relevant to food and feed safety, on account of their toxic effects and abundance (Knutsen et al., 2018).

Several strategies have been considered to control pathogen infection and mycotoxin contamination in maize, both at pre- and postharvest stages (Palumbo et al., 2020; Logrieco et al., 2021), including the use of resistant cultivars, improved agronomic practices (Mesterházy et al., 2012), crop rotation, and fungicide and insecticide application (Folcher et al., 2009; Mazzoni et al., 2011; Alberts et al., 2016). Nevertheless, a strategy that could prevent FB contamination is not yet available. Therefore, breeding for mycotoxin resistance is so far considered as one of the most promising and sustainable approaches for preventing FB contamination in maize (Gaikpa and Miedaner, 2019; Mesterhazy et al., 2020).

Upon fungal infection, a multicomponent defense response is activated in maize (Lanubile et al., 2017). This response has been investigated over time using a number of different “omics” techniques, including next-generation sequencing (Lanubile et al., 2014; Wang et al., 2016; Stagnati et al., 2019), proteomics (Pechanova and Pechan, 2015), metabolomics (Wang et al., 2016; Ciasca et al., 2020), and lipidomics (Dall’Asta et al., 2015; Giorni et al., 2015; Righetti et al., 2019).

To date, metabolomics has been applied to the field of mycotoxin research, mainly to the *Fusarium graminearum*–wheat/barley system, with the aim to understand the role of trichothecenes, particularly deoxynivalenol, in pathogenesis (Gunnaiah and Kushalappa, 2014; Atanasova-Penichon et al., 2016; Richard-Forget et al., 2021). Only few studies have considered the *F. verticilliodes*–maize system using a targeted approach. These studies show that phenolic compounds, carotenoids, and phenylpropanoids are the major metabolite groups correlated with *F. verticillioides* pathogenesis in maize (Picot et al., 2013; Sampietro et al., 2013). Many of these compounds are responsible for cell-wall reinforcement, antimicrobial activity, apoptosis, and reactive oxygen species (ROS)-related process modulation.

Recent studies suggest a link between FB accumulation and the so-called plant lipid signature. The oxidation products of fatty acids, and the metabolic cascade thereof, seem to be involved in the host plant response. Some fatty acid hydroperoxides generated through the plant lipoxygenase pathways presumably mimic the fungal oxylipins, known as *psi*-factors, and are detected by the fungus, thus affecting mycotoxin biosynthesis and sporulation (Brodhagen and Keller, 2006; Kumari et al., 2011). Products derived from the action of 13-lipoxygenase (13-LOX) on linoleic acid repress the biosynthesis of aflatoxin, whereas those related to the 9-LOX pathway promote aflatoxin production (Burow et al., 1997).

The role of fungal linoleate diol synthase-coding gene, *lds-1*, and plant *lipoxygenase 3* coding gene, *LOX3*, has been investigated extensively, demonstrating that LOX3 is a major susceptibility factor in maize and is induced by fungal LDS1-derived oxylipins to suppress the jasmonate (JA) cascade (Scala et al., 2014, 2017; Battilani et al., 2018). In addition, the plant LOX3-mediated signaling promotes the biosynthesis of virulence-promoting oxylipins in the fungus. Therefore, diverse species of lipids and oxylipins has emerged as the key classes of compounds involved in the regulations of host–pathogen interactions.

Among the “omics” approaches, untargeted lipidomics coupled with a robust bioinformatic workflow, may serve as a powerful tool for the identification of biological pathways involved in plant–pathogen interaction. Although several attempts have been made to define the plant lipid signature using a targeted approach (Dall’Asta et al., 2015), only the use of a fully untargeted approach allowed for a more detailed picture (Righetti et al., 2019).

To date, plant resistance studies have preferentially been performed on inbred lines under environmentally controlled conditions. On the contrary, very few studies have focused on maize response to pathogens in the field, given the high variability in environmental conditions due to a number of co-occurring biotic and abiotic factors. Such conditions, although challenging, may offer a unique opportunity for exploring the real metabolic shift occurring in the plant upon fungal colonization under natural conditions (Giorni et al., 2015).

In our previous study, untargeted lipidomics was applied to a set of maize genotypes grown under open-field conditions in different geographical areas over a single harvest season (Righetti et al., 2019). Results showed that the hybrid genotype and environmental conditions have a significant influence on FB accumulation. Data analysis pointed out that the plant−pathogen interaction mainly affects glycerophospholipid and linoleic acid metabolic pathways. These findings suggested the involvement of oxylipin signaling as an early response in infected maize that precedes accumulation of FB. However, the preliminary study covered only 1year of observation in the field, and allowed us to annotate only 30 metabolites.

Starting from the previous basis, we designed a more robust open field study, considering two maize hybrids, with a different susceptibility toward FB_1_ accumulation, collected at two geographical areas over three harvest seasons. A comprehensive mapping of oxylipins and complex lipid signature was performed, and data were used for interpretation. This study aimed to further unravel the relevance of the biological pathways so far identified in the multifactorial maize response to GFSC infection, and to strengthen our comprehension of the molecular mechanisms underlying plant–pathogen interactions.

## Materials and Methods

### Chemicals and Reagents

Polytetrafluoroethylene (PTFE) 15-ml centrifugation cuvettes were obtained from Greiner Bio-One (Kremsmünster, Austria). HPLC grade methanol, ethanol, dichloromethane, 2-propanol, and hexane were purchased from Merck (Darmstadt, Germany). Ammonium formate and formic acid were purchased from Sigma-Aldrich (St. Louis, MO, United States). Water was purified using the Milli-Q purification system (Millipore, Bedford, MA, United States).

### Plant Material

Maize samples (*N*=84) were collected over 3years (2015, 2016, and 2017) in varietal field trials conducted in two northern Italian regions, Lombardia (*N*=42) and Piemonte (*N*=42), similarly to Righetti et al. (2019). Two mid-season hybrids, coded as H21 and H22 (FAO classes 500 and 600, respectively), were used in this study. The maize hybrid name was not reported because of the short market life of hybrids and the aim of collecting data not related to a specific hybrid or commercial brand but extendable to all hybrids (Dall’Asta et al., 2012). A fully balanced sampling was designed, with seven samples (*n*=7) for each hybrid at each location and at each harvest time, thus accounting for a total of 84 samples (*N*=7×2×2×3=84). For each sample, two biological replicates were considered accounting for the non homogeneous distribution of fumonisins (*N*_TOT_=84×3=252).

At maturity, ears were harvested manually from each 15-m^2^ plot (around 100 plants plot) to obtain a representative grain sample for the analyses. The ears were then dried at 60°C to approximately 14% humidity and shelled using an electric sheller. Kernels from each plot were mixed thoroughly to obtain a random distribution and then ground using a ZM 200 Ultra Centrifugal Mill (Retsch GmbH, Haan, Germany) fitted with a 1-mm sieve. The resulting whole maize meal was stored at 4°C until needed for subsequent experiments.

A representative subset of maize samples (five samples per geographical area per year; *N*=5×2×3=30) was selected for lipidomic analysis.

### Fumonisin Determination

Fumonisins determination was performed according to our previous well-established protocol (Dall’Asta et al., 2012), further adapted over time following instrumental advances, and recently described in Rabaaoui et al. (2021). Briefly, 2g of ground maize sample was mixed with 8ml of water: methanol (30:70, v/v) in an Ultraturrax T25 high-speed blender (IKA, Stauffen, Germany) at 6,000rpm for 3min. The resulting homogenous mixture was filtered through 0.45-μm nylon filters, and 1ml of the filtrate was analyzed by LC-electrospray ionization (ESI)-MS/MS. The total FB content was expressed as the sum of FB_1_, FB_2_, and FB_3_.

The analysis was carried out using an UHPLC Dionex Ultimate 3000 separation system coupled to a triple quadrupole mass spectrometer (TSQ Vantage; Thermo Fisher Scientific Inc., San Jose, CA, United States) equipped with an electrospray source (ESI). For the chromatographic separation, XBridge Amide BEH column (Waters, Wilmslow, United Kingdom) with 2.10×100mm and a particle size of 2.6μm heated to 40°C was used. The technical and quality parameters are reported in Rabaaoui et al. (2021), and are therefore given as Supplementary Material.

Occurrence data for FB_1_, FB_2_, and FB_3_ are given as mean±SD in the Supplementary Material (Supplementary Table S1).

### Untargeted Lipidomics

#### Sample Preparation and Detection

Maize samples were ground into a fine powder using a ball mill (MM 301 Retsch, Haan, Germany). Then, 1g of the ground maize sample was extracted with 10ml of dichloromethane: methanol (50:50, v/v) first by manually shaking the sample for 1min and subsequently using an automatic shaker (IKA Laboratortechnik, Germany) at 240 strokes/min for 30min. Maize extracts were then centrifuged at 13,416×*g* for 5min at 20°C (Rotina 35 R, Hettich Zentrifugen, Germany). Prior to analysis, 1ml of the extract was evaporated under a gentle stream of nitrogen gas, and the residue was reconstituted in 1ml of 2-propanol: methanol: water mixture (65:30:5, v/v/v).

Lipidomics analysis was carried out using ultra-high performance liquid chromatography coupled with quadrupole-time-of-flight mass spectrometry (UHPLC-Q-TOF-MS), as described previously (Righetti et al., 2019). Briefly, the chromatographic separation of the extract was performed using 1290 UHPLC system (Agilent Technologies, Santa Clara, CA, United States), equipped with BEH C_18_ analytical column (2.1mm×100mm, 1.7μm) maintained at 60°C. The mobile phase was a binary mixture of solvent A [5mM ammonium formate and 0.1% formic acid in water: methanol (95:5, v/v)] and solvent B [5mM ammonium formate and 0.1% formic acid in 2-propanol: methanol: water (65:30:5, v/v/v)]. A multi-step elution dual-mode gradient was optimized from 10 to 100% B over a 17.5-min run. The sample injection volume was 1μl, and the autosampler temperature was maintained at 5°C. Untargeted acquisition was performed using 6550 iFunnel QTOF mass spectrometer detector (Agilent Technologies, Santa Clara, CA, United States) with positive polarity (scan, 100–1,000*m/z* range at a rate of 0.8 spectra/s) and extended dynamic range mode.

To perform tandem MS spectra acquisition, a data-dependent Auto MS/MS method was employed. Product ion spectra (*m/z* range=50–1,200), were acquired for the 10 most intensive ions in each survey spectrum, with the following parameters: collision energy=35V; collision energy spread=±15V.

The in-batch sequence of samples was random (established on the basis of random number generation) to avoid any possible time-dependent changes during UHPLC-Q-TOF-MS analysis, which could result in false clustering. Each set of samples was preceded by three blank controls: Milli-Q water, methanol, and no sample. The quality control (QC) sample, prepared by pooling an aliquot of the extract from each sample, was injected at the beginning of the sequence and after every 10 sample injections.

Details of acquisition parameters have been reported previously (Righetti et al., 2019).

#### Data Processing and Chemometrics Analysis

Fumonisin-target quantification data were statistically analyzed using multi-factor ANOVA (MANOVA), followed by Tukey’s *post hoc* test (*α*=0.05), using the year, the hybrid, and the harvesting area as factors. The analysis was carried out on the full dataset (*N*=252) using Statistica 13 (TIBCO Software Inc., Paolo Alto, CA, United States).

Raw untargeted lipidomic data were deconvoluted using the Profinder B.07 software (Agilent Technologies). The expansion of values for chromatogram extraction was set to 10ppm, and single-ion identification was excluded. Data pre-processing (mass and retention time alignment and compound filtering), normalization, and baselining were conducted in Profinder B.07 (Righetti et al., 2019).

The filtered dataset was then exported into MetaboAnalyst 4.0 (Chong et al., 2019), log-transformed, and Pareto-scaled before evaluating the quality of the unsupervised and supervised models. Principal component analysis (PCA) was performed to assess natural sample grouping. Significant variables were selected, according to the volcano plot (fold change>2; FDR corrected value of *p*<0.01).

Compound annotation was carried out using the Profinder B.07 software, based on the “find-by-formula” algorithm. Putative identification was achieved based on the database exported from METLIN,^1^ KEGG,^2^ LIPID MAPS,^3^ and using the entire isotopic profile (monoisotopic mass, isotope spacing, and ratio), with a maximum mass accuracy of 5ppm for each centroid mass in the isotopic cluster. To achieve a higher degree of confidence in annotation, a further identification/confirmation step was carried out from QCs in MS-DIAL 3.98 (Tsugawa et al., 2015). This step was performed using publicly available MS/MS experimental spectra built into the MassBank of North America (MoNA) repository and MS-Finder in-silico fragmentation data of compounds in Lipid Maps and PlantCyc (Tsugawa et al., 2016). Therefore, annotation was carried out according to “level III” (putatively characterized compounds) and when possible “level II” (putatively identified compounds), corresponding to compounds identified by UHPLC–HRMS/MS, and matched to spectra from databases and literature, as set out by the Metabolomic Standard Initiative (MSI Board Members et al., 2007).

Differential compounds were subjected to fold-change analysis by comparing the H21 and H22 groups, and then uploaded into the Omics Viewer pathway tool of PlantCyc^4^ to identify pathways and processes from the metabolite list (Paley et al., 2017).

## Results and Discussion

### Fumonisin Accumulation

All samples were found to be fumonisin-contaminated, with the sum of FB_1_, FB_2_, and FB_3_ ranging from the limit of detection (LOD: 10μg/kg) to 32,386μg/kg. The results are reported in the Supplementary Material (values are given as mean and SD). We investigated the effects of the three main factors (harvest season, geographical area, and hybrid) on FB_1_ and total FBs, because FB_2_ and FB_3_ are less abundant than FB_1_ ant their amount is strictly correlated with FB_1_. For statistical calculation, the full dataset was used, considering each biological replicate as a single datapoint (*N*_TOT_=252).

The level of FB_1_ accumulation varied significantly with all three factors (year, *p*<0.000; area, *p*<0.000; and hybrid, *p*=0.017), whereas total FBs accumulation varied significantly with the geographical area (*p*<0.000) and harvest year (*p*<0.000; Table 1). These results are consistent with previous studies and our preliminary study (Righetti et al., 2019) showing the strong effect of environmental factors (mainly related to climate conditions) on FBs contamination. However, it must be noted that the hybrid as well as its interaction with the harvest year were found to exert significant effects on FBs contamination of maize in the current study.

**Table 1:** Multi-factor ANOVA (MANOVA) analysis performed on fumonisin accumulation in hybrid maize samples grown in different geographical regions over 3years (full dataset, *N*_TOT_=252).

		Total FBs^†^		Fumonisin B_1_ (FB_1_)^†^	
		Mean concentration (μg/kg)	Range (μg/kg)	Mean concentration (μg/kg)	Range (μg/kg)
Year	2015	9,256b	79–32,702	7,089a	LOD–19,637
	2016	5,309b	LOD–26,871	4,073b	LOD–22,250
	2017	10,558a	1,459–26,435	7,921a	727–21,060
	*value of p*	< 0.000		< 0.000	
Area	LM	11,549a	255–32,702	8,829a	433–22,250
	PM	5,245b	LOD–21,892	3,861b	LOD–19,448
	*value of p*	< 0.000		< 0.000	
Hybrid	H21	8,588		6,596a	209–22,250
	H22	8,371		6,263b	LOD–17,967
	*value of p*	0.061		0.017	
year*area	*value of p*	0.181		0.001	
year*hybrid	*value of p*	0.104		0.004	
area*hybrid	*value of p*	0.224		0.176	
area*hybrid*year	*value of p*	0.231		0.189	

† *Different lowercase letters indicate significant differences (p<0.05)*.

*n.s., non-significant; LOD, limit of detection, 10μg/kg*.

### Lipidomics Analysis

Given the large variability in FB accumulation over years and across different geographical areas, we selected a representative subset of maize samples for untargeted lipidomics analysis, with the identification of plant biochemical pathways affected by FBs as the main goal. The samples were tagged according to the hybrid code (H21 vs. H22), and the range and median values of FB_1_ concentration were calculated for each group, as follows: H21, median=14,054μg/kg (range: 4,175–21,416μg/kg); H22, median=779μg/kg (range: LOD–6,293μg/kg). Since *F. verticillioides* infection has not been measured in the present study, we cannot directly correlate the FBs accumulation to the infection rate. However, we can argue that a low FBs accumulation in H22 was due to a lower infection rate or to a lower ability of the fungus to produce FBs in the host. It must be underlined that the lower FBs accumulation in H22 was conserved in the open field over three harvest years and two geographical areas. Therefore, we could reasonably argue that H22 can be regarded as a more resistant hybrid than H21.

Samples were analyzed by UHPLC-Q-TOF-MS under a fully untargeted approach using the same conditions as reported in our previous work (Righetti et al., 2019) to allow data comparison. The raw data were subjected to PCA to obtain an overview of the trend in an unsupervised manner and to determine putative outliers. After a quality assessment, the data were filtered by choosing entities present with a rate of 100% in at least one sample group, resulting in a reduced dataset of 2,940 features.

**Figure 1:**
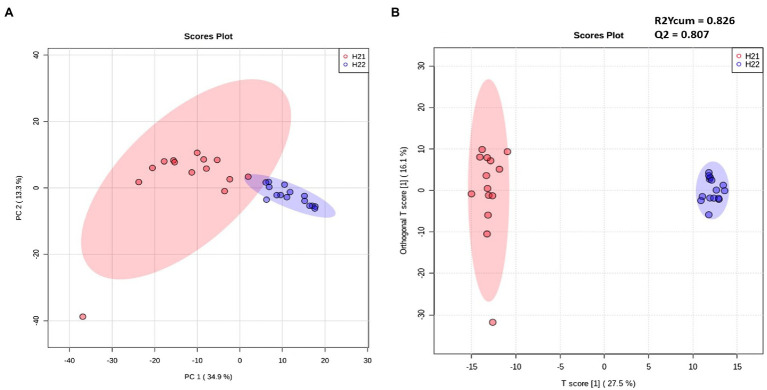
Unsupervised (left) and supervised (right) statistical models of H21 and H22 samples. **(A,B)** Principal component analysis (PCA) score plot **(A)** and Orthogonal Partial Least-Square Discriminant Analysis (OPLS-DA; *R*^2^_ycum_=0.826, *Q*^2^=0.807) score plot **(B)** constructed using filtered data and colored according to the level of fumonisin contamination. Red and blue colors represent H21 and H22 samples, respectively.

Samples were divided into two major groups, according to the contamination level, indicating a correlation between FB accumulation and the maize lipidome signature (Figure 1A). Samples from the H22 group were randomly arranged over the 95% confidence ellipse, whereas the H21 samples were tightly clustered. No clusters were detected for the harvest year or geographical area (Supplementary Figure S1). Although our lipidomic approach covered only a small portion of the whole plant metabolome, clear differences between the two groups were evident, suggesting the strong involvement of the lipid fraction in the response to FB accumulation in the two hybrids.

**Figure 2:**
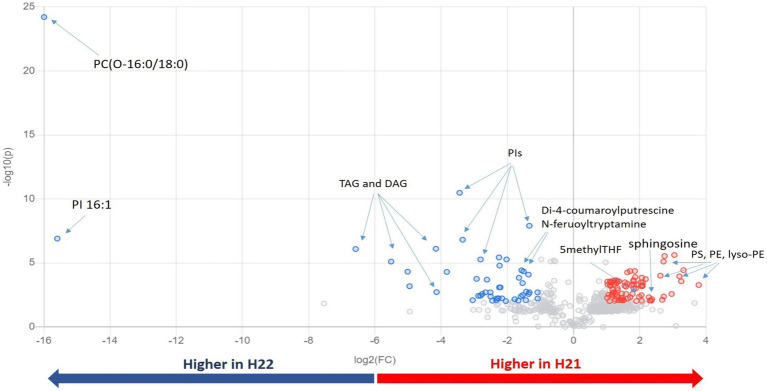
Important features selected by volcano plot analysis, based on the thresholds of fold change [*x*-axis; log_2_(FC)=2] and *t*-test [*y*-axis; −log_10_(*p*)=0.01]. Data were adjusted according to the false discovery rate (FDR). Colored circles represent features above the thresholds; features accumulated to higher levels in H21 are indicated in red, whereas those accumulated to higher levels in H22 are indicated in blue. The further its position from the (0,0) point, the more significant the feature. PC, glycerophosphocholine; PE, glycerophosphoethanolamine; PG, glycerophosphoglycerol; PI, glycerophosphoinositol; TAGs, triacylglycerols; DAGs, diacylglycerols; and THF, tetrahydrofolate.

Next, we inspected and validated the supervised models and then discriminated the metabolites based on a combination of value of *p* (<0.05) and fold-change (FC>2). The supervised clusterization is reported in Figure 1B. Overall, 361 significant metabolites were annotated; the tentative identification, pseudomolecular ion, retention time, and composite spectrum of these metabolites are summarized in Supplementary Material and Supplementary Table S2. Volcano plot analysis (Figure 2) showed that glycerolipids, such as glycerophosphoethanolamine (PE), glycerophosphoserine (PS), and their lyso-forms were higher in the resistant H21 hybrid than in H22 samples; whereas glycerophosphoinositol (PI) and glycerophosphocholine (PC) were lower in H21 than in H22 samples. Compared with H22 samples, sphingoid bases and folate-related compounds were higher in H21, whereas hydroxycinnamate amides such as feruoyltryptamine and di-4-coumaroylputrescine were less abundant in H21 samples.

### Functional Enrichment and Pathway Analysis

To further identify metabolic pathways involved in the plant response associated with FB contamination, we performed functional enrichment analysis using PlantCyc. The most significant pathways (*p*≤0.01) are listed in Table 2; these included pathways involved in the biosynthesis of lipids, such as glycerophospholipids, glycerolipids (diacylglycerol and triacylglycerol), and fatty acids.

**Table 2:** List of metabolic pathways involved in the response of maize plants to fumonisin accumulation.

Pathway	*p*
Superpathway of phospholipid biosynthesis	1.6E-07
Diacylglycerol and triacylglycerol biosynthesis	1.6E-06
Choline biosynthesis	3.5E-05
Amine and polyamine biosynthesis	3.8E-05
Fatty acid and lipid degradation	4.1E-05
Phosphatidylethanolamine biosynthesis	9.5E-05
Phosphatidylserine biosynthesis	7.8E-04
Sphingolipid biosynthesis	9.6E-04

To identify metabolic pathways differentially modulated between H21 and H22, leading to differential accumulation of FBs at harvest, we performed compound annotation and enrichment analysis, following data processing. The results showed that oxidized fatty acids, oxylipins, sphingolipids, and glycerophospholipids were the main lipid classes disrupted by fungal infection (discussed below).

#### Oxylipins

Lipid peroxidation is known to be a key factor in the regulation of mycotoxin production (Fabbri et al., 1983). Through the formation of oxygenated fatty acids or oxylipins, ROS are responsible for the activation of the plant defense frontline during the infection process (Scala et al., 2014; Oldenburg et al., 2017).

In the current study, several 9- and 13-oxylipins as well as oxidized fatty acid precursors were annotated. However, none of these compounds showed a statistical significance in sample clusterization, according to the volcano plot. The lack of significance of oxylipins may be ascribable to the fact that oxylipins spike following infection is expected at an early stage upon seed development; while at harvest they could have been already catabolized.

**Figure 3:**
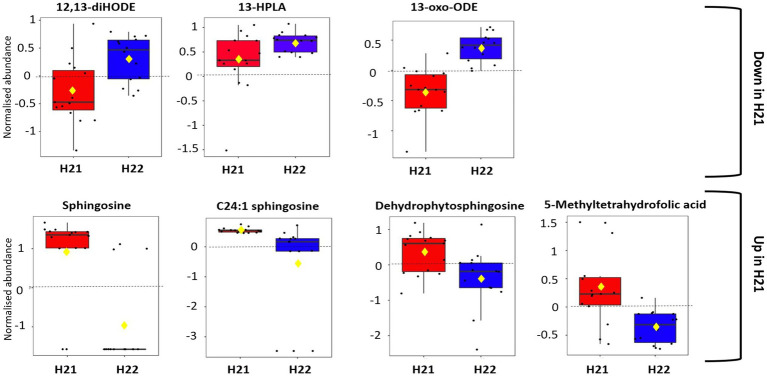
Boxplot analysis of selected metabolites. Black dots represent the normalized abundance of selected features in all samples. The notch indicates 95% CI around the median of each group, defined as ±1.58*IQR/sqrt(n). The mean concentration is indicated with a yellow diamond.

Although a change (decrease or increase) in metabolite abundance only by a factor of 2 was considered significant in this study, the data on 13-oxylipin accumulation can be interpreted in terms of tendency. We found that 13-oxylipins, such as 12,13-diHODE, 13-oxoODE, and 13(S)-hydroperoxylinolenic acid (13-HPLA), were less abundant in H21 than in H22 samples (Figure 3), suggesting that when FBs are accumulated to high levels in maize following pathogen infection, 13-oxylipins are consumed by the plant to activate the JA signaling pathway, as reported previously (Mosblech et al., 2009; Wasternack and Feussner, 2017). Although the biological relevance to resistance against *F. verticillioides* is currently unclear, based on our data, we can suggest a regulatory role of 13-oxylipins in the considered pathosystem. Because our lipidomic analytical conditions were not suitable for JA analysis, this hypothesis deserves further investigation. However, it must be noted that 13-oxylipins are involved in the regulation of fungal growth and mycotoxin production in *Aspergillus* (Deboever et al., 2020), and fungal oxylipins are able to hijack plant oxylipin processes to facilitate the onset of disease and the production of mycotoxins (Gao and Kolomiets, 2009). Unfortunately, we were unable to map significant changes in the 9-oxylipin pathway. In particular, 9-HODE has been acknowledged as a mycotoxin-susceptibility factor (Scala et al., 2014). However, it must be noted that C18:2 and C18:3 derived oxylipins are linked to early FBs accumulation stages as recently reported by several authors (Christensen and Kolomiets, 2011; Dall’Asta et al., 2012; Beccaccioli et al., 2021).

The oxylipin cascade is known as a non-stress-specific signaling pathway activated by the plant under biotic and abiotic stress conditions (Wasternack and Feussner, 2017). Therefore, many different stressors may stimulate the oxylipin signaling pathway in maize grown in the open field, regardless of GFSC infection and FB accumulation. Notwithstanding, oxylipin signatures are reported to change from the early to late phase of plant response and to exhibit different dynamics as a function of both the organ considered and the senescence status of those organs (Ghanem et al., 2012; Muñoz and Munné-Bosch, 2020). As reported previously, several oxylipins are produced by both the fungus and the plant. The extent at which each organism genotype determines the pattern of specific oxylipin accumulation, is strongly affected by the stage of disease progression (Battilani et al., 2018). In the present study, seeds at the harvest stage were considered for fingerprinting, and this could have prevented the detection of significant changes in the oxylipin profile. Therefore, to better understand the events occurring in the field, the maize metabolic fingerprint should be further studied at different time points after fungal infection when seeds are not matured yet and they are metabolically responsive to infection.

Furthermore, it must be considered that plants adopt efficient processes to prevent the accumulation of peroxidation products, as these could potentially damage cellular membranes. Hydroperoxides, a peroxidation product, are cleaved into volatile aldehydes and an oxo fatty acid by hydroperoxide lyase, rather than being metabolized further by divinyl ether synthase and allene oxide synthase and other sub-branches of the LOX pathway (Mosblech et al., 2009). In parallel, non-enzymatic chemical reactions involving singlet oxygen and other ROS also occur and contribute to the oxylipin signature by producing hydroperoxides, phytoprostanes, hydroxyalkenals, and ketols (Mosblech et al., 2009). Nonetheless, oxylipins have been reported to be esterified by a number of other molecules in the cell (Mosblech et al., 2009). This intricate set of diverse enzymatic and non-enzymatic reactions produces metabolites not detected by mass spectrometric analyses and undergoes dynamic changes in response to the progression of fungal infection and leaf senescence, as well as in response to different environmental conditions (Blée, 2002). In addition, it must be noted that oxylipins may be esterified to complex membrane lipids, which were not measured in this study. Taking this information into account, it is not surprising that oxylipins did not show significant differences in our study, where a number of diverse factors were included in the experimental design to mimic real-case scenarios. In fact, oxylipins are biochemical intermediates, and therefore they can be easily converted into different forms as an effect of environmental factors (Eckardt, 2008; Hou et al., 2016).

#### Sphingolipids and Glycerophospholipids

Sphingolipids are well-known to function as anchors for membrane proteins (Futerman, 1995), as well as secondary messengers for multiple cellular functions (Berkey et al., 2012; Beccaccioli et al., 2019). Sphingolipids are formed by the reduction and subsequent N-acetylation of long-chain sphingoid bases, such as sphingosine, and may further react to form complex sphingolipids, including inositol phosphorylceramides and glucosylceramides. Ceramide synthase (CerS) catalyzes the N-acetylation of sphingoid bases, also known as a long-chain base (LCB; Merrill et al., 2015). The class is usually grouped according to the length of the fatty acid chain, being the long-chain fatty acids (LCFAs) those with 12–18 carbon atoms (C12–C18), and the very LCFAs (VLCFAs) those with 20–30 carbon atoms (C20–C36). It has been demonstrated that CerS activity, is strongly disrupted by FB_1_.

It has been recently demonstrated that, among CerS isoforms, maize homologs of CerS2 are preferentially targeted by FBs, increasing therefore the formation of LCFA-sphinganine structures over VLCFA-phytosphingosine ones (Zitomer et al., 2008; Beccaccioli et al., 2021). Very recently, Beccaccioli et al. (2021) suggested that FB-driven massive increase in free sphingoid bases and S-LCFA may represent a key signal for controlling programmed cell death in maize infected seedlings.

In the present study, free sphingoid bases were more abundant in H21 than in H22 maize samples (Figure 3). This is in agreement with the disruption of CerS by FBs and the subsequent reduction in phytoceramide synthesis in highly contaminated samples, as previously reported by many authors (Zitomer et al., 2008; Dall’Asta et al., 2015; Beccaccioli et al., 2021). In agreement with Beccaccioli et al. (2021), we observed an accumulation of S-LCFA in highly contaminated samples, thus confirming the preferential disruption of CerS2 homologs in maize.

Because of the disruption of sphingolipid biosynthesis, the metabolism of glycerophospholipids was also altered in H21 samples, consistent with previous studies (Rubert et al., 2017; Righetti et al., 2019). Glycerophospholipids (GPLs), phosphate-containing glycerol-based lipids, are the major components of cellular membranes, thus constituting the structural barrier of cells. GPL species are involved in multiple membrane-associated processes, such as membrane trafficking and signal transduction (Meijer and Munnik, 2003). The generation of different GPL classes is strongly interconnected as well as connected to the production of diacylglycerides. Because GPLs are involved in hormone signaling and cell membrane function, their metabolism is strongly affected by pathogen infection. This is consistent with our results, which showed a significant disruption of all phospholipid classes upon FB accumulation.

Lyso-glycophospholipids are generated by the selective cleavage of oxidized fatty acid chains from GPLs (Soo et al., 2014). The removal of oxidized fatty acids from the phospholipid backbone may reflect metabolite rearrangement for cellular signaling to either overcome cell damage or trigger oxylipin biosynthesis in response to FB contamination. In the current study, lyso-forms of all GPL species were found in both H21 and H22 samples. The higher abundance of GPL lyso-forms in H21 samples may be connected to greater turnover of oxylipin biosynthesis as a consequence of higher FB accumulation.

Among all GPL species, PIs were particularly abundant in H22 compared with H21 samples. These molecules play a crucial role in the plant-pathogen interaction, inducing ROS production as a defense response (Gonorazky et al., 2008). PI have been reported to also play a role in pathogen plant cell internalization (Yaeno et al., 2011) and in activating resistance genes related to sialic acid (Antignani et al., 2015). The higher PI abundance in the susceptible H21 may be ascribable to higher activation of plant defense strategies to counteract higher FB accumulation. Furthermore, considering that PI-derived precursors act as important regulators of multiple processes during plant growth and development (Meijer and Munnik, 2003), the relatively low PI content in H21 samples may suggest an impairment of growth-related processes at higher FB concentration.

#### Other Pathways

**Figure 4:**
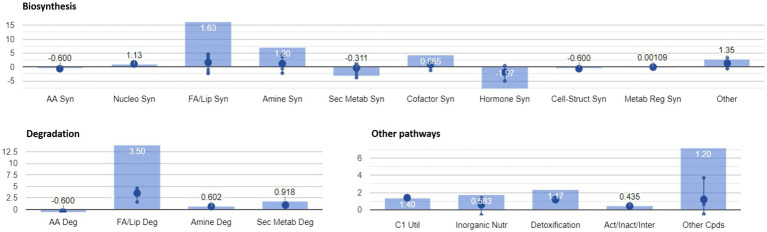
Identification of plant metabolic processes impaired by FB accumulation. Differential pathways and their fold-change (FC) values were determined using the Omics Viewer Dashboard of the PlantCyc Pathway Tool software. Large dots represent the mean of all log_2_(FC) values of the pathways, and small dots represent the log_2_(FC) values of different biochemical classes of metabolites included in a specific pathway. The *x*-axis represents each set of subcategories, while the *y*-axis corresponds to the cumulative log_2_(FC). The value of *p* is reported in white at the top of each bar. Syn, biosynthesis; Deg, degradation; FA/Lip, fatty acids and lipids; Amines, amines and polyamines; Sec Metab, secondary metabolites synthesis; AA, amino acids; Nucleo, nucleotides; Cell-Struct, compounds responsible for cell structure; C1 util, consumption of carbon sources; Act/Inact/Inter, activation, inactivation, and interaction pathways; and Cpds, compounds.

**Figure 5:**
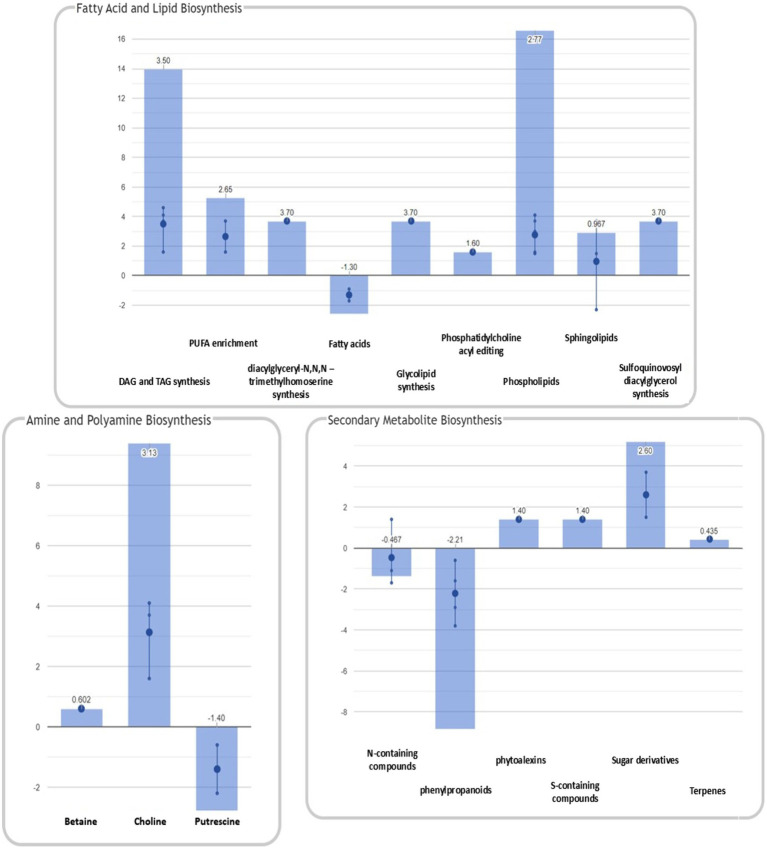
Summary of plant metabolic pathways impaired by FB accumulation. Differential pathways and their FC values were determined using the Omics Viewer Dashboard of the PlantCyc Pathway Tool software. Large dots represent the mean of all log_2_(FC) values of the pathways, and small dots represent the log_2_(FC) values of different biochemical classes of metabolites included in a specific pathway. The *x*-axis represents each set of subcategories, while the *y*-axis corresponds to the cumulative log_2_(FC). The value of *p* is reported in white at the top of each bar.

In addition to the pathways reported above, enrichment analysis confirmed the significant modulation of pathways identified in our previous study (Righetti et al., 2019). A graphical representation of the overall metabolic processes impaired upon FB accumulation is depicted in Figure 4, while a more detailed representation of the involved pathways is shown in Figure 5.

The activation of both synthesis and degradation pathways suggests that compounds are synthesized to be readily channeled into another target pathway. In the case of fatty acids and lipids, their production and simultaneous degradation can be related to the activation of the signaling cascade (i.e., oxylipins including the defense hormone JA). Polyamines are known to play important roles in almost all environmental stresses, including salt, drought, extreme temperature, flooding, heavy metal, acidic pH, and oxidative stresses (Shi and Chan, 2014). The biosynthesis and degradation of polyamine were mirrored in a general depletion of amino acid related metabolism (mean log_10_FC=−0.600). Interestingly, the “C1 pool by folate” pathway was activated in H21 samples. In agreement with our previous study (Righetti et al., 2019), 5-methyltetrahydrofolic acid was accumulated to significantly higher levels in samples with high FB contamination than in those with low levels of contamination. Folates act as both donors and acceptors of one-carbon groups in one-carbon transfer reactions, supporting the regulation of gene expression as well as the synthesis of lipids, proteins, chlorophyll, and lignin. In plants, folates are involved in ROS-mediated oxidation and cellular respiration. Recently, the negative effect of the folate metabolic pathway on plant immunity was elucidated in *Arabidopsis thaliana* (Gonzalez and Vera, 2019). The detoxification pathway was also activated in H21 compared with the H22 hybrid, with the degradation of ROS driving the pathway (mean log_10_FC=1.17).

A general activation of fatty acid and lipid biosynthesis (FA/lip syn; mean log_10_FC=1.63) and degradation (FA/lip deg.; mean log_10_FC=3.50) was observed in H21 samples compared with H22. Similarly, amine biosynthesis (mean log_10_FC=1.20) and degradation (mean log_10_FC=0.602) were increased in H22 samples, with the activation of choline and betaine synthesis and the putrescine pathway.

A general decrease in secondary metabolite biosynthesis (mean log_10_FC=−0.311), together with an increase in secondary metabolite degradation (mean log_10_FC=0.918), was observed in H21 samples compared with H22. However, when the effect on each pathway is observed independently (Figure 5), it can be noticed that only the phenylpropanoid biosynthesis pathway was strongly downregulated in H21 samples compared with H22. Given that phenylpropanoids are involved in cell wall reinforcement (Miedes et al., 2014; Lanubile et al., 2017), our results can explain the higher FB accumulation in H21 hybrid compared with H22. However, further studies should be carried out to carefully map the phenylpropanoid profile and confirm this preliminary observation.

## Conclusion

To the best of our knowledge, this is the first comprehensive attempt to map the lipidomic response of maize hybrids to FB_1_ accumulation under real open field conditions and over 3years of observation.

Altogether, the results of this study demonstrate that the strong activation of the lipid signaling cascade in the presence of FB_1_ already seen in controlled experiments occurs under open field conditions as well. The activation of such a frontline plant defense machinery may derive from a direct induction following fungal infection in the field, leading to the elicitation of a non-specific lipid-based plant response toward biotic and abiotic stressors. Because this study was carried out under open field conditions, we could not determine whether the direct induction was exerted by FB_1_ itself or by other fungal metabolites synthesized at the infection stage. It must be stressed out the *F. verticillioides* infection rate was not determined in H21 and H22 in this study. Reasonably, the infection rate in H22 was lower than in H21, and this might be caused by the higher activation of lipid signaling systems observed in H22. Since the role played by FB_1_ in eliciting the plant lipid signature remains unclear, future studies are needed to better understand the triggering phase of the plant–pathogen interaction. However, our observation collected in an open field study, are of upmost relevance to validate and connect the biological evidence returned by model systems to the real agronomic conditions.

Considering that H21 and H22 samples were exposed to the same environmental factors (i.e., location and harvest season), the higher activation of lipid signaling systems in H22 suggests that other routes are enabled in the less susceptible hybrids to limit FBs accumulation. Despite the evidence supporting the role of the hybrid in limiting FBs accumulation in our study, the genotype-driven specific resistance factors likely act by limiting the initial event (i.e., fungal infection) and are therefore no longer detectable at the harvest stage.

To elucidate the metabolic pathways involved in specific resistance responses, susceptible and resistant maize hybrids should be further compared in time-course experiments. Comparing the metabolome fingerprint of maize hybrids at the initial fungal infection event, and subsequently during the growth season, will help map the modulation of different metabolic routes over time.

## Data Availability Statement

The original contributions presented in the study are included in the article/Supplementary Material, further inquiries can be directed to the corresponding authors.

## Author Contributions

PB and CD’A conceived the study. LR carried out the metabolomics analysis and data analysis. LL supervised the analytical part and contributed to the interpretation of results. LR and CD’A contributed to the writing process. PB and LL critically revised the final manuscript. PB supervised the project and provided funding. All authors provided critical feedback and helped to shape the research. All authors contributed to the article and approved the submitted version.

## Funding

This work was supported by funding from the Italian Ministry of Agricultural Food and Forestry and Tourism Policies (MiPAAFT; to PB) as part of the following research program: RQC MAIS (D.D.N.88666 03/12/2014).

## Conflict of Interest

The authors declare that the research was conducted in the absence of any commercial or financial relationships that could be construed as a potential conflict of interest.

## Publisher’s Note

All claims expressed in this article are solely those of the authors and do not necessarily represent those of their affiliated organizations, or those of the publisher, the editors and the reviewers. Any product that may be evaluated in this article, or claim that may be made by its manufacturer, is not guaranteed or endorsed by the publisher.

## Footnotes

^1^ http://metlin.scripps.edu

^2^ http://www.genome.jp/kegg/genome.html

^3^ https://lipidmaps.org/

^4^ https://plantcyc.org/
