# Supplementary material for: Lipid Signaling Modulates the Response to Fumonisin Contamination and Its Source, Fusarium verticillioides, in Maize
Source: Front Plant Sci. 2021 Nov 8;12:701680. doi: 10.3389/fpls.2021.701680 (PMC8606633; doi:10.3389/fpls.2021.701680)
Supplement: Supplementary file 1 [file Data_Sheet_1.docx]

Supplementary Material

Lipid signaling modulates the response to fumonisin contamination and its source, *Fusarium verticillioides*, in maize

Laura Righetti^1^, Chiara Dall’Asta^1*^, Luigi Lucini^2^, Paola Battilani^3*^

^1^Department of Food and Drug, University of Parma, Parma, Italy

^2^Department for Sustainable Food Process, Università Cattolica del Sacro Cuore, Piacenza, Italy

^3^Department of Sustainable Crop Production, Università Cattolica del Sacro Cuore, Piacenza, Italy

*** Correspondence:**Chiara Dall’Asta
chiara.dallasta@unipr.it

Paola Battilani
paola.battilani@unicatt.it

**Analytical conditions for fumonisin quantification.**

The analysis was carried out using an UHPLC Dionex Ultimate 3000 separation system coupled to a triple quadrupole mass spectrometer (TSQ Vantage; Thermo Fisher Scientific Inc., San Jose, CA, USA) equipped with an electrospray source (ESI). For the chromatographic separation, XBridge Amide BEH column (Waters, Wilmslow, UK) with 2.10 x 100 mm and a particle size of 2.6 µm heated to 40 °C was used.

Gradient elution was performed by using acetonitrile (eluent A), water acidified with 0.2% formic acid (eluent B) and ammonium formate 20 mM 1% formic acid (eluent C). The elution gradient started from 0% of B, 5% of C and 95% of A and, after an initial isocratic step of 2 min, B increased at 60% in 3 min and this composition is kept for 2.5 min. At 8 min, the initial conditions were restored and kept for 5 min. The total run time was 13 min.

A thorough in-house validation procedure was performed during method development, and regularly verified over time. Recovery was between 89% – 93% for fumonisins, with a repeatability ranging between 5 – 10%. Matrix-matched calibration curves (calibration range, 25−5000 μg/kg) were used for the quantification. The limit of quantification (LOQ) was 25 μg/kg, and the LOD was found to be lower than 10 μg/kg for all of the considered analytes. All of the results were corrected for recovery. Samples showing contamination levels higher than the highest calibration level (5000 μg/kg) were diluted to match the proper calibration range.

**Supplementary Table S1: Occurrence data obtained for the sample set over three years of observation (data are reported as mean concentration ± SD).**

| **Year** | **Code** | **Hybrid** | **Area** | **FB1**  **(µg/Kg)** | **FB2**  **(µg/Kg)** | **FB3**  **(µg/Kg)** |
| --- | --- | --- | --- | --- | --- | --- |
| 2015 | AGR15 - 197 | H21 | LM | 19611 ± 37 | 2254± 18 | 814±27 |
| 2015 | AGR15 - 204 | H21 | LM | 15974 ± 1356 | 3789 ± 207 | 2130±121 |
| 2015 | AGR15 - 212 | H21 | LM | 18828 ± 69 | 4326 ± 60 | 2271±211 |
| 2015 | AGR15 - 219 | H21 | LM | 7779 ± 193 | 4384 ± 20 | 1155±89 |
| 2015 | AGR15 - 258 | H21 | LM | 2849 ± 104 | 1423 ± 17 | 287±11 |
| 2015 | AGR15 - 265 | H21 | LM | 3648± 325 | 334± 6 | 160±12 |
| 2015 | AGR15 - 273 | H21 | LM | 1142 ± 34 | 240 ± 8 | 109±21 |
| 2015 | AGR15 - 033 | H21 | PM | 487 ± 77 | 67±11 | 32±2 |
| 2015 | AGR15 - 040 | H21 | PM | 1473 ± 4 | 1170 ± 2 | 95±6 |
| 2015 | AGR15 - 055 | H21 | PM | 1118 ± 54 | 1156 ± 12 | 79±11 |
| 2015 | AGR15 - 002 | H21 | PM | 6387 ± 192 | 612 ± 0 | 268±22 |
| 2015 | AGR15 - 008 | H21 | PM | 9384 ± 164 | 766 ± 10 | 267±32 |
| 2015 | AGR15 - 015 | H21 | PM | 4139 ± 123 | 1223 ± 43 | 718±34 |
| 2015 | AGR15 - 022 | H21 | PM | 17478±692 | 1814±180 | 795±28 |
| 2015 | AGR15 - 005 | H22 | PM | 2235 ± 112 | 1343 ± 27 | 192±12 |
| 2015 | AGR15 - 011 | H22 | PM | 17031 ± 210 | 4689 ± 32 | 214±9 |
| 2015 | AGR15 - 019 | H22 | PM | 831 ± 39 | 225 ± 0 | 91±2 |
| 2015 | AGR15 - 209 | H22 | LM | 19193 ± 360 | 13193 ± 87 | 2117±34 |
| 2015 | AGR15 - 214 | H22 | LM | 852 ± 10 | 4690 ± 32 | 1980±34 |
| 2015 | AGR15 - 222 | H22 | LM | 5677 ± 103 | 1367 ± 16 | 541±22 |
| 2015 | AGR15 - 053 | H22 | PM | 46 ± 25 | 57 ± 8 | 30±1 |
| 2015 | AGR15 - 257 | H22 | LM | 7151 ± 86 | 4850 ± 19 | 766±21 |
| 2015 | AGR15 - 269 | H22 | LM | 210 ± 1 | 50 ± 6 | LOD |
| 2015 | AGR15 - 027 | H22 | PM | 9813 ± 301 | 2810 ± 99 | 1213±34 |
| 2015 | AGR15 - 029 | H22 | PM | 905 ± 6 | 1101 ± 3 | LOD |
| 2015 | AGR15 - 039 | H22 | PM | 120 ± 9 | 59 ± 1 | LOD |
| 2015 | AGR15 - 261 | H22 | LM | 903 ± 0 | 1092 ± 2 | LOD |
| 2015 | AGR15 - 276 | H22 | LM | 7033 ± 158 | 1613 ± 44 | 888±44 |
| 2016 | AGR16-19 | H21 | LM | 2469 ± 256 | 1331 ± 56 | 386±21 |
| 2016 | AGR16-26 | H21 | LM | 4175 ± 102 | 2612 ± 12 | 410±12 |
| 2016 | AGR16-43 | H21 | LM | 21416 ± 1179 | 4323 ± 421 | 1125±32 |
| 2016 | AGR16-70 | H21 | LM | 9401± 250 | 516± 23 | 413±21 |
| 2016 | AGR16-77 | H21 | LM | 4682 ± 66 | 1001± 11 | 760±43 |
| 2016 | AGR16-05 | H21 | LM | 2750 ± 145 | 1410 ± 28 | 428±17 |
| 2016 | AGR16-12 | H21 | LM | 1692 ± 205 | 355 ± 38 | 234±27 |
| 2016 | AGR16-162 | H21 | PM | 2796 ± 137 | 1091 ± 17 | 320±21 |
| 2016 | AGR16-257 | H21 | PM | 6293 ± 500 | 2139 ± 101 | 38±1 |
| 2016 | AGR16-264 | H21 | PM | 2242 ± 154 | 737 ± 55 | 121±9 |
| 2016 | AGR16-277 | H21 | PM | 1081 ± 24 | 1080 ± 6 | LOD |
| 2016 | AGR16-284 | H21 | PM | 1380 ± 110 | 1138 ± 14 | 72±2 |
| 2016 | AGR16-291 | H21 | PM | 970 ± 29 | 1066 ± 9 | LOD |
| 2016 | AGR16-298 | H21 | PM | 1136 ± 131 | 1104 ± 23 | 63±2 |
| 2016 | AGR16-18 | H22 | LM | 1057 ± 7 | 215 ± 8 | 141±11 |
| 2016 | AGR16-31 | H22 | LM | 8012 ± 240 | 1379 ± 43 | 971±21 |
| 2016 | AGR16-37 | H22 | LM | 1395 ± 38 | 311 ± 13 | 177±12 |
| 2016 | AGR16-44 | H22 | LM | 2566 ± 42 | 584 ± 38 | 382±15 |
| 2016 | AGR16-57 | H22 | LM | 12258 ± 273 | 2697 ± 93 | 804±23 |
| 2016 | AGR16-01 | H22 | LM | 6673 ± 198 | 1950 ± 188 | 480±26 |
| 2016 | AGR16-08 | H22 | LM | 5491 ± 123 | 1292 ± 56 | 209±9 |
| 2016 | AGR16-253 | H22 | PM | 3443 ± 91 | 1885 ± 21 | 713±17 |
| 2016 | AGR16-258 | H22 | PM | 674 ± 12 | 206 ± 8 | 79±3 |
| 2016 | AGR16-266 | H22 | PM | 471 ± 15 | 116 ± 6 | LOD |
| 2016 | AGR16-275 | H22 | PM | 1125 ± 27 | 1140 ± 4 | LOD |
| 2016 | AGR16-282 | H22 | PM | LOD | LOD | LOD |
| 2016 | AGR16-289 | H22 | PM | 612 ± 17 | 702 ± 4 | LOD |
| 2017 | AGR17 -091 | H21 | LM | 12134 ± 750 | 2638 ± 251 | 1743±131 |
| 2017 | AGR17 -112 | H21 | LM | 11427 ± 318 | 4322 ± 46 | 1396±87 |
| 2017 | AGR17 -098 | H21 | LM | 20810 ± 355 | 4954 ± 21 | 1376±71 |
| 2017 | AGR17 -105 | H21 | LM | 17973 ± 81 | 3954 ± 2 | 1548±62 |
| 2017 | AGR17 -148 | H21 | PM | 2195 ± 118 | 629 ± 24 | 248±37 |
| 2017 | AGR17 -155 | H21 | PM | 1221 ± 45 | 1486 ± 16 | 144±12 |
| 2017 | AGR17 -141 | H21 | PM | 1356 ± 10 | 999 ± 11 | 107±11 |
| 2017 | AGR17 -162 | H21 | PM | 1019 ± 2 | 946 ± 19 | 44±6 |
| 2017 | AGR17 -175 | H21 | PM | 7425 ± 165 | 2561 ± 58 | 889±17 |
| 2017 | AGR17 -196 | H21 | PM | 8908 ± 210 | 2832 ± 19 | 921±15 |
| 2017 | AGR17 -182 | H21 | PM | 4560 ± 386 | 1412 ± 144 | 1043±54 |
| 2017 | AGR17 -197 | H21 | LM | 2665 ± 239 | 1111 ± 28 | 202±73 |
| 2017 | AGR17 -204 | H21 | LM | 4568 ± 377 | 1383 ± 52 | 441±11 |
| 2017 | AGR17 -211 | H21 | LM | 4973 ± 281 | 1519 ± 16 | 481±32 |
| 2017 | AGR17 -236 | H21 | LM | 9280 ± 175 | 2175 ± 317 | 1207±32 |
| 2017 | AGR17 -206 | H22 | LM | 15066 ± 894 | 2908 ± 212 | 1159±141 |
| 2017 | AGR17 -106 | H22 | LM | 14395 ± 376 | 6586 ± 146 | 2079±178 |
| 2017 | AGR17 -093 | H22 | LM | 7914 ± 505 | 2402 ± 349 | 1852±99 |
| 2017 | AGR17 -234 | H22 | LM | 18172 ± 171 | 6785 ± 319 | 2010±162 |
| 2017 | AGR17 -242 | H22 | LM | 9290 ± 265 | 6244 ± 38 | 1285±87 |
| 2017 | AGR17 -228 | H22 | LM | 10808 ± 232 | 3524 ± 76 | 1043±75 |
| 2017 | AGR17 -249 | H22 | LM | 8835 ± 61 | 2877 ± 38 | 1027±78 |
| 2017 | AGR17 -149 | H22 | PM | 2932 ± 76 | 1232 ± 4 | 439±12 |
| 2017 | AGR17 -158 | H22 | PM | 1737 ± 85 | 1161 ± 56 | 168±11 |
| 2017 | AGR17 -142 | H22 | PM | 728 ± 1 | 732 ± 1 | 39±4 |
| 2017 | AGR17 -174 | H22 | PM | 7532 ± 350 | 1857 ± 2 | 899±18 |
| 2017 | AGR17 -195 | H22 | PM | 7480 ± 345 | 2812 ± 80 | 821±21 |
| 2017 | AGR17 -179 | H22 | PM | 2160 ± 44 | 640 ± 12 | 1143±43 |
| 2017 | AGR17 -188 | H22 | PM | 12145 ± 272 | 3776 ± 56 | 1411±42 |

**Supplementary Table S2: Annotation of the significant features obtained for the H21 *versus* H22 sample set**

| Class | Compound name | Reference m/z | Rt  (min) | log2(FC) | Annotation level | Annotation score | MS isotopic spectrum |
| --- | --- | --- | --- | --- | --- | --- | --- |
| 2-prenylated xanthones | 1-Hydroxy-3,6,7-trimethoxy-2-(3-methyl-2-butenyl)-8-(3-hydroxy-3-methyl-1E-butenyl)-xanthone | 455.2139 | 4.6 | 0.9 | MS/MS in silico prediction | 99.4 | 455.21423:825012 456.21758:267384 457.22094:45447 |
| Acetogenines | Montecristin | 575.4993 | 20.1 | 0.8 | MS/MS in silico prediction | 100 | 575.49921:2980546 576.50256:1204201 577.50592:1760779 |
| Acetogenines | Cohibin C | 577.5150 | 20.9 | 1.0 | MS/MS in silico prediction | 100 | 577.51508:1739677 578.51843:717570 579.52179:157874 |
| Amino acids and derivatives | Aspartyl-Glutamine | 244.0945 | 3.1 | -1.3 | MS-only | 96.9 | 244.09088:9302 245.09423:0 246.09759:0 |
| Amino acids and derivatives | L-Arginine | 175.1180 | 1.4 | -0.6 | MS/MS in silico prediction | 98.9 | 175.1171:535502 176.12045:68242 177.12381:11327 |
| Amino acids and derivatives | Betaine | 118.0849 | 1.6 | 0.9 | MS/MS in silico prediction | 99.1 | 118.08469:7006302 119.08804:384660 120.0914:35273 |
| Amino acids and derivatives | N,N-Dimethyl-L-proline | 144.1007 | 5.6 | 0.9 | MS/MS in silico prediction | 99.5 | 144.10063:53244 145.10398:7866 146.10734:9444 |
| Amino acids and derivatives | Carnitine | 162.1125 | 1.4 | 1.0 | MS-only | 99.2 | 162.1107:341193 163.11405:39156 164.11741:0 |
| Amino acids and derivatives | Glutathione (oxidized) | 613.1592 | 6.0 | 1.4 | MS-only | 97.1 | 613.16266:34123 614.16601:14146 615.16937:0 |
| Bioactives | Thiamine | 265.1130 | 8.6 | -1.7 | MS/MS in silico prediction | 95.7 | 265.10892:6869 266.11227:0 267.11563:6910 |
| Bioactives | 5-Methyltetrahydrofolic acid | 442.1837 | 3.2 | 0.9 | MS-only | 96.6 | 442.18741:14536 443.19076:0 444.19412:0 |
| Coumaric acids and derivatives | Hexadecyl ferulate | 419.3137 | 12.1 | -0.6 | MS/MS in silico prediction | 99.8 | 419.31366:42963 420.31701:16704 421.32037:12852 |
| Diacylglycerols | DAG(16:0/18:1(9Z)/0:0) | 577.5155 | 15.0 | -8.6 | MS/MS in silico prediction | 99.9 | 577.51538:10753525 578.51873:6888324 579.52209:1502676 |
| Diacylglycerols | DAG(18:1(11Z)/18:1(9Z)/0:0) | 603.5321 | 15.1 | -7.5 | MS/MS in silico prediction | 99.7 | 603.53119:12136905 604.53454:8368465 605.5379:1970532 |
| Diacylglycerols | DAG 67:10; DAG 23:0-44:10 | 1056.9318 | 20.9 | -3.9 | MS-only | 90.7 | 1056.93811:11694 1057.94146:9833 1058.94482:6910 |
| Diacylglycerols | DAG 47:1; DAG 9:0-38:1 | 794.7596 | 24.5 | -2.5 | MS-only | 99.2 | 794.76074:7211 795.76409:0 796.76745:0 |
| Diacylglycerols | DAG 22:0; DAG 8:0-14:0 | 446.3840 | 17.6 | -2.2 | MS-only | 99.5 | 446.38434:6517 447.38769:0 448.39105:0 |
| Diacylglycerols | DAG 43:6e; DAG 15:0e/28:6 | 714.6395 | 22.3 | -1.8 | MS-only | 96.8 | 714.64276:6229 715.64611:0 716.64947:0 |
| Diacylglycerols | DAG 48:8e; DAG 20:1e/28:7 | 780.6865 | 16.3 | -1.8 | MS-only | 99.7 | 780.68707:56958 781.69042:39573 782.69378:22255 |
| Diacylglycerols | DAG 38:7; DAG 8:0-30:7 | 656.5248 | 14.9 | -0.8 | MS-only | 99.8 | 656.52515:72015 657.5285:28862 658.53186:0 |
| Diacylglycerols | DAG 47:6; DAG 9:0-38:6 | 784.6813 | 14.9 | -0.8 | MS-only | 97 | 784.67786:10718 785.68121:6835 786.68457:18606 |
| Diacylglycerols | DAG 41:1; DAG 9:0-32:1 | 710.6657 | 16.1 | -0.7 | MS-only | 93.1 | 710.66034:56251 711.66369:34062 712.66705:0 |
| Diacylglycerols | DAG 36:1e; DAG 8:0e/28:1 | 626.6082 | 15.2 | -0.6 | MS-only | 91.8 | 626.60229:16468 627.60564:7848 628.609:0 |
| Diacylglycerols | DAG 66:6; DAG 22:0-44:6 | 1050.9788 | 23.2 | -0.5 | MS-only | 87.9 | 1050.97144:23239 1051.97479:17780 1052.97815:11264 |
| Diacylglycerols | DAG 44:4; DAG 8:0-36:4 | 746.6657 | 13.8 | 0.2 | MS-only | 95.6 | 746.66998:39576 747.67333:26450 748.67669:9468 |
| Diacylglycerols | DAG 49:5e; DAG 21:0e/28:5 | 800.7490 | 17.0 | 0.6 | MS-only | 93.1 | 800.74365:32818 801.747:21310 802.75036:65756 |
| Diacylglycerols | DAG 37:6; DAG 9:0-28:6 | 644.5248 | 13.8 | 0.6 | MS-only | 98.4 | 644.52722:20097 645.53057:7983 646.53393:0 |
| Diacylglycerols | DAG 56:9e; DAG 28:2e/28:7 | 890.7960 | 20.7 | 0.7 | MS-only | 93.3 | 890.80127:281027 891.80462:71854 892.80798:25773 |
| Diacylglycerols | DAG 56:14; DAG 18:5-38:9 | 894.6970 | 19.9 | 0.7 | MS-only | 99.6 | 894.69672:7096 895.70007:128118 896.70343:73780 |
| Diacylglycerols | DAG 54:12e; DAG 26:5e/28:7 | 856.7177 | 19.1 | 0.7 | MS-only | 99.7 | 856.71753:4818 857.72088:0 858.72424:0 |
| Diacylglycerols | DAG 34:8; DAG 16:3-18:5 | 598.4466 | 12.9 | 0.7 | MS-only | 84.7 | 598.45508:109724 599.45843:73504 600.46179:35541 |
| Diacylglycerols | DAG 64:12; DAG 20:0-44:12 | 1010.8535 | 16.1 | 0.8 | MS-only | 99.9 | 1010.85297:8945 1011.85632:5654 1012.85968:0 |
| Diacylglycerols | DAG 56:8e; DAG 28:1e/28:7 | 892.8117 | 20.0 | 0.8 | MS-only | 99.6 | 892.81238:38925 893.81573:0 894.81909:0 |
| Diacylglycerols | DAG 70:18; DAG 26:6-44:12 | 1082.8535 | 17.5 | 0.9 | MS-only | 99.7 | 1082.85449:9256 1083.85784:8737 1084.8612:0 |
| Diacylglycerols | DAG 37:3e; DAG 9:0e/28:3 | 636.5925 | 14.3 | 1.0 | MS-only | 92.6 | 636.58691:479261 637.59026:275666 638.59362:73610 |
| Diacylglycerols | DAG 56:8; DAG 12:0-44:8 | 906.7909 | 16.0 | 1.2 | MS-only | 99.9 | 906.79065:20054 907.794:14070 908.79736:16278 |
| Diacylglycerols | DAG 43:3e; DAG 15:0e/28:3 | 720.6865 | 17.0 | 1.2 | MS-only | 89.7 | 720.67975:7147 721.6831:0 722.68646:0 |
| Diacylglycerols | DAG 56:6e; DAG 28:0e/28:6 | 896.8430 | 20.9 | 1.2 | MS-only | 97.8 | 896.84015:10695 897.8435:0 898.84686:0 |
| Diacylglycerols | DAG 29:1e; DAG 8:0e/21:1 | 528.4987 | 14.3 | 1.3 | MS-only | 91.6 | 528.49268:10779 529.49603:0 530.49939:0 |
| Diacylglycerols | DAG 44:8; DAG 8:0-36:8 | 738.6031 | 11.1 | 1.3 | MS-only | 89 | 738.59607:37849 739.59942:27183 740.60278:8948 |
| Diacylglycerols | DAG 64:14; DAG 20:2-44:12 | 1006.8222 | 24.6 | 1.3 | MS-only | 99.2 | 1006.82104:10598 1007.82439:5463 1008.82775:0 |
| Diacylglycerols | DAG 43:9e; DAG 17:2e/26:7 | 708.5925 | 16.1 | 1.3 | MS-only | 99.4 | 708.59137:13067 709.59472:9714 710.59808:0 |
| Diacylglycerols | DAG 44:4e; DAG 16:0e/28:4 | 732.6865 | 17.0 | 1.4 | MS-only | 91.8 | 732.68054:71488 733.68389:49122 734.68725:18387 |
| Diacylglycerols | DAG 45:4; DAG 9:0-36:4 | 760.6813 | 15.5 | 1.4 | MS-only | 91.4 | 760.67523:17244 761.67858:12198 762.68194:10554 |
| Diacylglycerols | DAG 65:14; DAG 21:2-44:12 | 1020.8378 | 26.2 | 1.6 | MS-only | 99.3 | 1020.8371:22376 1021.84045:9749 1022.84381:0 |
| Diacylglycerols | DAG 44:3e; DAG 16:0e/28:3 | 734.7021 | 17.2 | 1.7 | MS-only | 94.5 | 734.69733:9824 735.70068:7982 736.70404:0 |
| Diacylglycerols | DAG 45:6e; DAG 17:0e/28:6 | 742.6708 | 15.4 | 1.8 | MS-only | 99.2 | 742.67236:12146 743.67571:7935 744.67907:13569 |
| Diacylglycerols | DAG 42:3e; DAG 14:0e/28:3 | 706.6708 | 16.9 | 1.9 | MS-only | 93.8 | 706.66571:21964 707.66906:16781 708.67242:0 |
| Diacylglycerols | DAG 42:2e; DAG 14:0e/28:2 | 708.6865 | 17.4 | 2.1 | MS-only | 92.4 | 708.68079:27296 709.68414:19202 710.6875:6173 |
| Diacylglycerols | DAG 47:8e; DAG 19:1e/28:7 | 766.6708 | 16.5 | 2.1 | MS-only | 95.6 | 766.66656:307802 767.66991:211303 768.67327:132291 |
| Diacylglycerols | DAG 47:7; DAG 9:0-38:7 | 782.6657 | 14.6 | 2.1 | MS-only | 99.8 | 782.6651:22400 783.66845:14231 784.67181:14348 |
| Diacylglycerols | DAG 31:2; DAG 9:0-22:2 | 568.4935 | 15.6 | 2.2 | MS-only | 96.9 | 568.49011:7576 569.49346:0 570.49682:0 |
| Diacylglycerols | DAG 56:10; DAG 12:0-44:10 | 902.7596 | 24.0 | 3.7 | MS-only | 99.5 | 902.75952:10839 903.76287:5603 904.76623:0 |
| Fatty acyl carnitines | ACar 25:6 | 514.3891 | 13.3 | -3.0 | MS-only | 96.5 | 514.39276:5191 515.39611:0 516.39947:0 |
| Fatty acyl carnitines | O-(17-carboxyheptadecanoyl)carnitine | 458.3447 | 8.2 | -0.9 | MS/MS in silico prediction | 99.6 | 458.34424:520292 459.34759:163589 460.35095:37083 |
| Fatty acyl carnitines | ACar 23:5 | 488.3734 | 8.9 | -0.6 | MS-only | 95.2 | 488.3779:86468 489.38125:0 490.38461:6258 |
| Fatty acyl carnitines | ACar 26:1 | 538.4830 | 14.2 | 0.6 | MS-only | 99.8 | 538.48273:9676 539.48608:0 540.48944:0 |
| Fatty acyl carnitines | ACar 8:2 | 284.1856 | 5.2 | 0.6 | MS-only | 89.9 | 284.17892:24337 285.18227:11229 286.18563:0 |
| Fatty acyl carnitines | ACar 24:5 | 502.3891 | 8.6 | 1.3 | MS-only | 94.2 | 502.39404:13141 503.39739:5279 504.40075:19516 |
| Fatty acyl carnitines | Stearoylcarnitine | 410.3265 | 6.2 | 1.8 | MS-only | 99.9 | 410.32715:29632 411.3305:13404 412.33386:15895 |
| Fatty amides | Lignoceroyl-ethanolamine | 412.4223 | 8.2 | -1.7 | MS/MS in silico prediction | 99.6 | 412.42273:70710 413.42608:26996 414.42944:0 |
| Flavanoids | 5'-Hydroxy-3',4',7,8-tetramethoxyflavan | 329.1234 | 9.7 | -2.8 | MS/MS in silico prediction | 99.7 | 329.12372:12019 330.12707:0 331.13043:0 |
| Flavanoids | 2',7-Dihydroxy-4'-methoxy-8-prenylflavan 2',7-diglucoside | 647.2803 | 1.7 | -2.7 | MS-only | 96.4 | 647.27643:14306 648.27978:7527 649.28314:0 |
| Flavanoids | (3R)-7,2',4'-Trihydroxy-8,3'-diprenyloxyisoflavan | 395.2306 | 14.6 | -1.2 | MS-only | 93 | 395.23584:6950 396.23919:0 397.24255:0 |
| [Flavonoids](http://classyfire.wishartlab.com/tax_nodes/C0000334) | 3,5,6-Trihydroxy-3',4',7-trimethoxyflavone | 361.0918 | 4.7 | -3.8 | MS/MS in silico prediction | 99.4 | 361.09186:39956 362.09521:11169 363.09857:0 |
| Flavonoids | Artonin E | 437.1598 | 1.6 | -0.8 | MS/MS in silico prediction | 98.3 | 437.1579:1318216 438.16125:264815 439.16461:0 |
| Flavonoids | 4'-Hydroxy-5,7,2'-trimethoxyflavanone 4'-rhamnosyl-(1->6)-glucoside | 671.2595 | 1.6 | 2.1 | MS/MS in silico prediction | 96 | 671.25592:27653 672.25927:11079 673.26263:0 |
| Flavonoids | 3',8-Dihydroxy-4',5',7-trimethoxyflavone | 345.0972 | 5.5 | -2.9 | MS/MS in silico prediction | 99.5 | 345.09723:1850563 346.10058:486999 347.10394:181436 |
| Flavonoids | (S)-5,7-Dihydroxy-6-prenylflavanone | 307.1417 | 5.5 | -2.8 | MS/MS in silico prediction | 99.5 | 307.14178:3065962 308.14513:762072 309.14849:101505 |
| Flavonoids | 4',5-Dihydroxy-3',5',7,8-tetramethoxyflavone | 375.1075 | 5.4 | -2.3 | MS/MS in silico prediction | 99.5 | 375.10742:2632800 376.11077:645967 377.11413:262812 |
| Glycerophosphocholines | PC 62:13 | 1128.8355 | 9.8 | -4.3 | MS-only | 98.2 | 1128.83813:10249 1129.84148:12285 1130.84484:0 |
| Glycerophosphocholines | PC 53:11 | 1028.7079 | 7.7 | -3.2 | MS-only | 97 | 1028.70435:4169 1029.7077:0 1030.71106:205852 |
| Glycerophosphocholines | PC 48:10 | 960.6453 | 4.3 | -2.3 | MS-only | 97.6 | 960.64215:11478 961.6455:0 962.64886:0 |
| Glycerophosphocholines | PC(44:1) | 956.8218 | 16.2 | -1.8 | MS-only | 99.4 | 956.82031:386656 957.82366:280926 958.82702:125111 |
| Glycerophosphocholines | LysoPC 30:3 | 708.4938 | 8.3 | -0.8 | MS-only | 94.1 | 708.48883:8775 709.49218:0 710.49554:0 |
| Glycerophosphocholines | PC 69:4 | 1267.0679 | 17.6 | -0.7 | MS-only | 99.9 | 1267.06763:183445 1268.07098:145598 1269.07434:71082 |
| Glycerophosphocholines | LysoPC 26:4e | 614.4544 | 9.9 | -0.7 | MS-only | 96 | 614.45844:8069 615.46179:31151 616.46515:18902 |
| Glycerophosphocholines | LysoPC 40:6 | 842.6034 | 17.4 | -0.7 | MS-only | 99.7 | 842.60376:17660 843.60711:8606 844.61047:0 |
| Glycerophosphocholines | LysoPC 42:7 | 868.6191 | 7.8 | -0.7 | MS-only | 97 | 868.61554:8118 869.61889:0 870.62225:0 |
| Glycerophosphocholines | PC 56:7 | 1078.8174 | 8.4 | -0.7 | MS-only | 92.4 | 1078.81165:599687 1079.815:481285 1080.81836:167954 |
| Glycerophosphocholines | LysoPC 30:0 | 714.5408 | 8.5 | -0.6 | MS-only | 85.9 | 714.53271:6299 715.53606:0 716.53942:0 |
| Glycerophosphocholines | PC(18:0/14:1(9Z)) | 732.5512 | 12.1 | -0.6 | MS/MS in silico prediction | 98 | 732.55396:6097 733.55731:0 734.56067:0 |
| Glycerophosphocholines | LysoPC 36:9 | 780.4938 | 7.9 | -0.5 | MS-only | 88.2 | 780.50116:13853 781.50451:7208 782.50787:0 |
| Glycerophosphocholines | PC 67:2 | 1243.0679 | 18.0 | -0.3 | MS-only | 99.7 | 1243.06689:74867 1244.07024:57145 1245.0736:25632 |
| Glycerophosphocholines | PC 65:4 | 1211.0053 | 17.6 | 0.2 | MS-only | 99.9 | 1211.00513:94412 1212.00848:81924 1213.01184:38989 |
| Glycerophosphocholines | PC 56:6e | 1044.8718 | 16.9 | 0.5 | MS-only | 99.9 | 1044.8717:21507 1045.87505:18993 1046.87841:7639 |
| Glycerophosphocholines | PC 54:5e | 1018.8562 | 17.1 | 0.6 | MS-only | 99.9 | 1018.85614:17732 1019.85949:17159 1020.86285:8669 |
| Glycerophosphocholines | LysoPC(24:0/0:0) | 590.4371 | 15.6 | 0.8 | MS/MS in silico prediction | 99.6 | 590.43591:13970 591.43926:6957 592.44262:0 |
| Glycerophosphocholines | LysoPC 29:0 | 700.5252 | 13.3 | 1.1 | MS-only | 82.2 | 700.51581:31727 701.51916:12677 702.52252:9642 |
| Glycerophosphocholines | PC 78:0 | 1379.2893 | 22.1 | 1.1 | MS-only | 99.5 | 1379.28833:14588 1380.29168:0 1381.29504:0 |
| Glycerophosphocholines | LysoPC 18:2 | 542.3217 | 11.1 | 1.2 | MS-only | 99.7 | 542.32202:12243 543.32537:8988 544.32873:16887 |
| Glycerophosphocholines | Lyso-PC 3:0e | 300.1570 | 4.7 | 1.3 | MS-only | 94.7 | 300.15231:270763 301.15566:62545 302.15902:9102 |
| Glycerophosphocholines | PC 54:4e | 1020.8719 | 17.5 | 1.4 | MS-only | 99.7 | 1020.87274:27702 1021.87609:24846 1022.87945:10981 |
| Glycerophosphocholines | Lyso-PC 30:8-SN1 | 676.4337 | 6.3 | 1.4 | MS-only | 99.8 | 676.43274:69451 677.43609:62298 678.43945:14371 |
| Glycerophosphocholines | Lyso-PC 28:5 | 676.4313 | 6.3 | 1.5 | MS-only | 97.5 | 676.4281:82640 677.43145:44540 678.43481:10275 |
| Glycerophosphocholines | Lyso-PC 5:0e | 328.1884 | 5.8 | 1.8 | MS-only | 96.2 | 328.18445:45257 329.1878:18243 330.19116:0 |
| Glycerophosphocholines | LysoPC 20:0 | 574.3843 | 9.7 | 1.8 | MS-only | 99.7 | 574.38348:9151 575.38683:13029 576.39019:7748 |
| Glycerophosphocholines | LysoPC 26:2 | 654.4469 | 14.5 | 2.1 | MS-only | 99.4 | 654.44574:9721 655.44909:0 656.45245:0 |
| Glycerophosphoethanolamines | PC(O-16:0/18:0) | 748.6215 | 13.2 | -16.0 | MS-only | 99.8 | 748.62067:12310 749.62402:0 750.62738:0 |
| Glycerophosphoethanolamines | LysoPE(20:5/0:0) | 500.2772 | 4.1 | -3.3 | MS-only | 91.4 | 500.27103:35887 501.27438:12431 502.27774:0 |
| Glycerophosphoethanolamines | LysoPE 18:1e | 466.3292 | 8.8 | -3.0 | MS-only | 95.6 | 466.32498:38235 467.32833:0 468.33169:0 |
| Glycerophosphoethanolamines | PE 42:2 | 828.6477 | 14.9 | -2.5 | MS-only | 99.8 | 828.64825:5818 829.6516:0 830.65496:7711 |
| Glycerophosphoethanolamines | PE 36:0 | 770.5670 | 13.0 | -2.5 | MS-only | 98 | 770.56421:73075 771.56756:51387 772.57092:15561 |
| Glycerophosphoethanolamines | PE 31:2 | 674.4755 | 13.3 | -2.3 | MS-only | 98.3 | 674.47803:25831 675.48138:13967 676.48474:5825 |
| Glycerophosphoethanolamines | PE 35:7 | 720.4599 | 3.4 | -2.2 | MS-only | 94.9 | 720.46454:6416 721.46789:0 722.47125:0 |
| Glycerophosphoethanolamines | PE 39:1e | 774.6372 | 18.5 | -1.9 | MS-only | 98.5 | 774.63495:8235 775.6383:0 776.64166:0 |
| Glycerophosphoethanolamines | LysoPE 15:1 | 438.2615 | 5.5 | -1.8 | MS-only | 87.1 | 438.26923:47200 439.27258:17010 440.27594:0 |
| Glycerophosphoethanolamines | PE 45:10 | 854.5695 | 13.9 | -1.7 | MS-only | 99.8 | 854.5694:27058 855.57275:19887 856.57611:11043 |
| Glycerophosphoethanolamines | PE 16:0 | 468.2721 | 4.6 | -1.5 | MS-only | 87.4 | 468.27969:57541 469.28304:21835 470.2864:0 |
| Glycerophosphoethanolamines | PE 64:16 | 1130.7549 | 7.7 | -1.4 | MS-only | 80.3 | 1130.76489:5639 1131.76824:0 1132.7716:0 |
| Glycerophosphoethanolamines | PE 52:11 | 972.6453 | 7.5 | -1.4 | MS-only | 88.9 | 972.63818:8821 973.64153:5222 974.64489:0 |
| Glycerophosphoethanolamines | PE 29:4 | 642.4129 | 8.0 | -1.3 | MS-only | 99.8 | 642.41229:7593 643.41564:5200 644.419:0 |
| Glycerophosphoethanolamines | PE 60:16 | 1074.6923 | 7.2 | -1.1 | MS-only | 85.1 | 1074.70068:7839 1075.70403:0 1076.70739:0 |
| Glycerophosphoethanolamines | PE 48:5 | 928.6766 | 9.3 | -1.0 | MS-only | 98 | 928.67371:17560 929.67706:7120 930.68042:0 |
| Glycerophosphoethanolamines | PE 31:1e | 662.5119 | 9.4 | -1.0 | MS-only | 99.9 | 662.51135:23237 663.5147:0 664.51806:0 |
| Glycerophosphoethanolamines | LysoPE 32:0 | 678.5432 | 12.0 | -0.9 | MS-only | 84.6 | 678.53467:24562 679.53802:11003 680.54138:5281 |
| Glycerophosphoethanolamines | LysoPE 28:7 | 608.3711 | 7.8 | -0.9 | MS-only | 88.3 | 608.37836:7907 609.38171:0 610.38507:0 |
| Glycerophosphoethanolamines | PE 43:7 | 854.5670 | 13.4 | -0.8 | MS-only | 99.4 | 854.56561:128520 855.56896:80371 856.57232:23906 |
| Glycerophosphoethanolamines | PE 74:15 | 1250.9451 | 8.6 | -0.7 | MS-only | 82.1 | 1250.95447:10531 1251.95782:10623 1252.96118:0 |
| Glycerophosphoethanolamines | PE 38:0 | 798.5983 | 13.7 | -0.7 | MS-only | 99.8 | 798.59875:19726 799.6021:12299 800.60546:9998 |
| Glycerophosphoethanolamines | PE 38:1 | 796.5827 | 13.2 | -0.7 | MS-only | 99.9 | 796.58301:44645 797.58636:28041 798.58972:13248 |
| Glycerophosphoethanolamines | PE 31:1 | 676.4912 | 8.0 | -0.7 | MS-only | 99.8 | 676.4903:7730 677.49365:0 678.49701:0 |
| Glycerophosphoethanolamines | PE 26:1 | 606.4129 | 8.1 | -0.7 | MS-only | 86.4 | 606.42084:5393 607.42419:0 608.42755:0 |
| Glycerophosphoethanolamines | PE 41:5e | 794.6058 | 9.0 | -0.6 | MS-only | 99.9 | 794.60547:12880 795.60882:8496 796.61218:0 |
| Glycerophosphoethanolamines | PE 39:4e | 768.5902 | 10.8 | -0.6 | MS-only | 89.5 | 768.59698:4885 769.60033:0 770.60369:0 |
| Glycerophosphoethanolamines | PE-NMe2(18:4)/14:0) | 712.4912 | 4.0 | -0.6 | MS-only | 92.3 | 712.49695:5226 713.5003:0 714.50366:0 |
| Glycerophosphoethanolamines | LysoPE 15:1e | 424.2823 | 4.1 | -0.6 | MS-only | 99.9 | 424.28156:6475 425.28491:0 426.28827:0 |
| Glycerophosphoethanolamines | LysoPE 34:3 | 700.5276 | 9.6 | -0.6 | MS-only | 99.9 | 700.52722:36349 701.53057:14588 702.53393:6149 |
| Glycerophosphoethanolamines | LysoPE 18:1(d7) | 487.3524 | 14.2 | -0.5 | MS-only | 99.9 | 487.35284:4777 488.35619:0 489.35955:0 |
| Glycerophosphoethanolamines | PE(18:2/P-18:1) | 726.5432 | 12.9 | -0.4 | MS-only | 93.3 | 726.5379:11730 727.54125:7829 728.54461:0 |
| Glycerophosphoethanolamines | N-arachidonoyl-PE(36:5) | 970.7471 | 21.4 | 0.7 | MS-only | 86.8 | 970.73938:17549 971.74273:6782 972.74609:0 |
| Glycerophosphoethanolamines | PE 61:5 | 1110.8800 | 18.2 | 0.8 | MS-only | 99.8 | 1110.87927:8938 1111.88262:7074 1112.88598:0 |
| Glycerophosphoethanolamines | PE 61:5 | 1088.8981 | 18.2 | 0.9 | MS-only | 99.9 | 1088.89771:10989 1089.90106:8707 1090.90442:0 |
| Glycerophosphoethanolamines | PE 37:7e | 734.5119 | 11.2 | 1.0 | MS-only | 99.7 | 734.51276:3718 735.51611:28040 736.51947:45926 |
| Glycerophosphoethanolamines | PE 45:2e | 856.7154 | 16.0 | 1.0 | MS-only | 98.6 | 856.71313:8981 857.71648:0 858.71984:0 |
| Glycerophosphoethanolamines | PE 53:5 | 976.7729 | 21.6 | 1.0 | MS-only | 99.5 | 976.77374:7972 977.77709:0 978.78045:0 |
| Glycerophosphoethanolamines | LysoPE 24:6 | 554.3241 | 9.1 | 1.0 | MS-only | 98.6 | 554.32635:25915 555.3297:10538 556.33306:0 |
| Glycerophosphoethanolamines | PE 55:0 | 1036.8644 | 16.3 | 1.0 | MS-only | 99.9 | 1036.86499:20914 1037.86834:11680 1038.8717:5028 |
| Glycerophosphoethanolamines | PE 55:1 | 1034.8488 | 17.4 | 1.0 | MS-only | 99.8 | 1034.84937:24625 1035.85272:19279 1036.85608:7783 |
| Glycerophosphoethanolamines | PE 51:2 | 976.7705 | 21.7 | 1.1 | MS-only | 99.5 | 976.77136:5884 977.77471:0 978.77807:0 |
| Glycerophosphoethanolamines | PE 45:0 | 874.7260 | 22.3 | 1.1 | MS-only | 99.5 | 874.72546:16802 875.72881:9450 876.73217:0 |
| Glycerophosphoethanolamines | PE 55:4 | 1028.8018 | 16.8 | 1.1 | MS-only | 99.5 | 1028.80042:6345 1029.80377:5381 1030.80713:6662 |
| Glycerophosphoethanolamines | PE 53:4e | 964.8093 | 17.5 | 1.2 | MS-only | 99.1 | 964.81104:38725 965.81439:31535 966.81775:11968 |
| Glycerophosphoethanolamines | PE 25:3 | 610.3479 | 1.7 | 1.2 | MS-only | 86.7 | 610.35577:660218 611.35912:360228 612.36248:74883 |
| Glycerophosphoethanolamines | PE 25:2 | 612.3636 | 1.6 | 1.2 | MS-only | 96.8 | 612.35992:4603 613.36327:0 614.36663:0 |
| Glycerophosphoethanolamines | LysoPE 20:2 | 506.3241 | 8.5 | 1.2 | MS-only | 96.4 | 506.32031:38691 507.32366:23570 508.32702:20681 |
| Glycerophosphoethanolamines | LysoPE 10:0e | 356.2196 | 4.7 | 1.2 | MS-only | 92.1 | 356.21378:113113 357.21713:29629 358.22049:6384 |
| Glycerophosphoethanolamines | PE 55:5 | 1004.8042 | 23.2 | 1.3 | MS-only | 99.6 | 1004.8045:10267 1005.80785:5796 1006.81121:0 |
| Glycerophosphoethanolamines | PE 35:3 | 750.5045 | 9.7 | 1.3 | MS-only | 98.5 | 750.50208:7481 751.50543:0 752.50879:0 |
| Glycerophosphoethanolamines | LysoPE 20:5e | 486.2979 | 9.2 | 1.3 | MS-only | 81.2 | 486.28827:8660 487.29162:0 488.29498:0 |
| Glycerophosphoethanolamines | PE 21:0 | 538.3503 | 10.2 | 1.3 | MS-only | 97.9 | 538.34753:42574 539.35088:31459 540.35424:9153 |
| Glycerophosphoethanolamines | PE 57:5 | 1032.8355 | 16.9 | 1.5 | MS-only | 99.9 | 1032.83557:90651 1033.83892:76201 1034.84228:30035 |
| Glycerophosphoethanolamines | PE 59:2 | 1088.8956 | 18.2 | 1.5 | MS-only | 99.6 | 1088.89673:9755 1089.90008:8672 1090.90344:5360 |
| Glycerophosphoethanolamines | LysoPE 22:3 | 532.3398 | 9.1 | 1.5 | MS-only | 91.7 | 532.34576:29086 533.34911:12568 534.35247:5764 |
| Glycerophosphoethanolamines | PE(52:4) | 986.7784 | 17.9 | 1.5 | MS-only | 97.8 | 986.77551:6345 987.77886:5807 988.78222:0 |
| Glycerophosphoethanolamines | PE 31:5 | 690.4105 | 6.6 | 1.6 | MS-only | 99.9 | 690.40991:26205 691.41326:15805 692.41662:0 |
| Glycerophosphoethanolamines | PE40:6 | 776.5589 | 13.4 | 1.6 | MS-only | 99.8 | 776.55865:69227 777.562:47322 778.56536:13455 |
| Glycerophosphoethanolamines | LysoPE 42:7 | 804.5902 | 15.8 | 1.9 | MS-only | 99.3 | 804.59149:4654 805.59484:0 806.5982:0 |
| Glycerophosphoethanolamines | PE 35:2 | 730.5382 | 13.6 | 1.9 | MS-only | 99.8 | 730.53821:49920 731.54156:36893 732.54492:15513 |
| Glycerophosphoethanolamines | LysoPE 38:4 | 754.5745 | 13.4 | 2.1 | MS-only | 99 | 754.57642:25836 755.57977:11538 756.58313:0 |
| Glycerophosphoethanolamines | LysoPE 40:8 | 774.5432 | 13.2 | 2.1 | MS-only | 99.7 | 774.54236:71691 775.54571:41133 776.54907:15963 |
| Glycerophosphoethanolamines | PE 49:4 | 944.7079 | 18.9 | 2.1 | MS-only | 99.5 | 944.70886:5704 945.71221:0 946.71557:0 |
| Glycerophosphoethanolamines | PE 55:4 | 1006.8198 | 24.5 | 2.1 | MS-only | 99.2 | 1006.81866:8552 1007.82201:0 1008.82537:0 |
| Glycerophosphoethanolamines | LysoPE 20:1 | 508.3398 | 8.9 | 2.3 | MS-only | 94.7 | 508.33511:25820 509.33846:11202 510.34182:8805 |
| Glycerophosphoethanolamines | PE 35:1 | 754.5357 | 13.2 | 2.3 | MS-only | 99.6 | 754.53461:32339 755.53796:18991 756.54132:21549 |
| Glycerophosphoethanolamines | PE 56:11e | 992.7466 | 21.2 | 2.7 | MS-only | 93.3 | 992.74146:16403 993.74481:10781 994.74817:0 |
| Glycerophosphoethanolamines | LysoPE 22:0 | 538.3867 | 10.3 | 2.7 | MS-only | 99.9 | 538.38666:34396 539.39001:15416 540.39337:0 |
| Glycerophosphoethanolamines | LysoPE(0:0/22:5) | 528.3024 | 8.5 | 2.7 | MS/MS in silico prediction | 99.7 | 528.30237:10525 529.30572:0 530.30908:13342 |
| Glycerophosphoethanolamines | LysoPE 42:0 | 818.6997 | 16.9 | 3.2 | MS-only | 99.8 | 818.69916:20986 819.70251:16895 820.70587:0 |
| Glycerophosphoglycerols | PG 44:8 | 892.6062 | 12.7 | -5.0 | MS-only | 99.8 | 892.60681:6767 893.61016:0 894.61352:0 |
| Glycerophosphoglycerols | PG(37:0) | 775.5748 | 12.6 | -2.6 | MS-only | 99.4 | 775.57611:5964 776.57946:5431 777.58282:0 |
| Glycerophosphoglycerols | PG(42:6) | 851.6038 | 8.1 | -2.0 | MS-only | 93.5 | 851.59851:6410 852.60186:0 853.60522:0 |
| Glycerophosphoglycerols | PG(O-16:0/21:0) | 801.5905 | 8.7 | -1.7 | MS-only | 97.8 | 801.59351:6516 802.59686:0 803.60022:0 |
| Glycerophosphoglycerols | PG 40:9 | 834.5280 | 12.2 | -1.6 | MS-only | 99.3 | 834.52942:9238 835.53277:5999 836.53613:0 |
| Glycerophosphoglycerols | PG 33:2 | 750.5280 | 7.9 | -1.4 | MS-only | 89 | 750.52094:5657 751.52429:5465 752.52765:0 |
| Glycerophosphoglycerols | PG 35:4 | 774.5280 | 8.3 | -0.9 | MS-only | 80.8 | 774.51813:36159 775.52148:11966 776.52484:0 |
| Glycerophosphoglycerols | PG(31:0) | 691.4810 | 11.4 | -0.8 | MS-only | 98 | 691.4837:22706 692.48705:13098 693.49041:0 |
| Glycerophosphoglycerols | PG 46:7 | 922.6531 | 8.3 | -0.5 | MS-only | 98.1 | 922.65594:8840 923.65929:5855 924.66265:0 |
| Glycerophosphoglycerols | PG 22:1 | 598.3715 | 8.7 | 1.0 | MS-only | 96.5 | 598.37518:185884 599.37853:61330 600.38189:13376 |
| Glycerophosphoglycerols | PG(33:1) | 717.4942 | 13.3 | 1.3 | MS-only | 99 | 717.49249:5673 718.49584:0 719.4992:0 |
| Glycerophosphoglycerols | PG(29:0) | 663.4473 | 9.7 | 1.4 | MS-only | 99.8 | 663.44781:12840 664.45116:6885 665.45452:0 |
| Glycerophosphoglycerols | PG 28:3 | 678.4341 | 8.5 | 1.4 | MS-only | 97.8 | 678.43701:26214 679.44036:16691 680.44372:5182 |
| Glycerophosphoglycerols | PG 26:5 | 646.3715 | 9.7 | 1.7 | MS-only | 98.1 | 646.37415:7414 647.3775:11799 648.38086:0 |
| Glycerophosphoglycerols | PG 20:1 | 570.3402 | 6.6 | 1.9 | MS-only | 97.4 | 570.33691:28439 571.34026:11744 572.34362:0 |
| Glycerophosphoglycerols | PG 40:2 | 848.6375 | 15.9 | 2.0 | MS-only | 99.7 | 848.63788:3663 849.64123:0 850.64459:0 |
| Glycerophosphoglycerols | PG(33:2) | 715.4810 | 13.7 | 2.6 | MS-only | 96.1 | 715.4848:23926 716.48815:0 717.49151:0 |
| Glycerophosphoglycerols | PG 40:10 | 832.5123 | 14.2 | 4.1 | MS-only | 97.3 | 832.5155:6224 833.51885:5115 834.52221:0 |
| Glycerophosphoinositols | PI 16:1 | 607.2490 | 3.8 | -15.3 | MS-only | 97.2 | 607.24567:10574 608.24902:6210 609.25238:0 |
| Glycerophosphoinositols | PI 15:1 | 593.2333 | 4.1 | -4.0 | MS-only | 99.3 | 593.23492:5127 594.23827:0 595.24163:0 |
| Glycerophosphoinositols | PI 42:2 | 969.6403 | 14.7 | -2.9 | MS-only | 99.9 | 969.6402:6951 970.64355:7429 971.64691:0 |
| Glycerophosphoinositols | PI 36:1 | 887.5620 | 15.5 | -2.4 | MS-only | 96.3 | 887.55823:44721 888.56158:38599 889.56494:11703 |
| Glycerophosphoinositols | PI 40:3 | 934.6379 | 12.7 | -2.3 | MS-only | 99.9 | 934.63776:10085 935.64111:7073 936.64447:0 |
| Glycerophosphoinositols | PI(36:0) | 849.5905 | 15.6 | -2.2 | MS-only | 99.6 | 849.591:8936 850.59435:6476 851.59771:0 |
| Glycerophosphoinositols | PI 41:7 | 940.5909 | 12.7 | -2.2 | MS-only | 91.4 | 940.59705:8050 941.6004:0 942.60376:0 |
| Glycerophosphoinositols | PI 44:4 | 988.6849 | 9.4 | -2.0 | MS-only | 99.8 | 988.68573:9149 989.68908:7781 990.69244:0 |
| Glycerophosphoinositols | PI 6:0 | 464.1527 | 3.0 | -1.6 | MS-only | 91.8 | 464.14676:107155 465.15011:28737 466.15347:6575 |
| Glycerophosphoinositols | PI 35:4 | 862.5440 | 7.8 | -1.6 | MS-only | 100 | 862.54413:6404 863.54748:0 864.55084:0 |
| Glycerophosphoinositols | PI 51:0 | 1099.8124 | 9.7 | -1.6 | MS-only | 99.8 | 1099.8114:12777 1100.81475:14271 1101.81811:0 |
| Glycerophosphoinositols | PI 4:0 | 441.0768 | 1.5 | -1.5 | MS-only | 82.1 | 441.08624:14518 442.08959:0 443.09295:0 |
| Glycerophosphoinositols | PI(38:2) | 873.5881 | 13.1 | -1.4 | MS-only | 98.7 | 873.59021:8918 874.59356:8632 875.59692:0 |
| Glycerophosphoinositols | PI 19:2 | 642.3249 | 4.1 | -1.3 | MS-only | 93.5 | 642.33014:7765 643.33349:0 644.33685:0 |
| Glycerophosphoinositols | PI 20:3 | 659.2803 | 5.0 | -1.3 | MS-only | 96.9 | 659.27673:13829 660.28008:7368 661.28344:0 |
| Glycerophosphoinositols | PI 31:0 | 814.5440 | 10.1 | -1.2 | MS-only | 96.5 | 814.54779:6167 815.55114:5521 816.5545:5513 |
| Glycerophosphoinositols | PI 5:0 | 450.1371 | 2.5 | -1.1 | MS-only | 98.7 | 450.1348:13124 451.13815:0 452.14151:0 |
| Glycerophosphoinositols | PI 25:4 | 722.3875 | 5.5 | -1.1 | MS-only | 99.1 | 722.38934:12292 723.39269:5774 724.39605:0 |
| Glycerophosphoinositols | PI 8:0 | 497.1394 | 3.7 | -0.9 | MS-only | 100 | 497.13943:14274 498.14278:0 499.14614:0 |
| Glycerophosphoinositols | PI 33:1 | 840.5596 | 7.6 | -0.8 | MS-only | 99.6 | 840.55835:12002 841.5617:6608 842.56506:0 |
| Glycerophosphoinositols | PI 41:4 | 946.6379 | 7.7 | -0.8 | MS-only | 99 | 946.63593:5424 947.63928:0 948.64264:0 |
| Glycerophosphoinositols | PI 17:0 | 623.2803 | 2.9 | -0.7 | MS-only | 85.2 | 623.27191:31599 624.27526:7953 625.27862:0 |
| Glycerophosphoinositols | PI 32:2 | 824.5284 | 7.8 | -0.7 | MS-only | 96.4 | 824.53223:10425 825.53558:6827 826.53894:0 |
| Glycerophosphoinositols | PI 41:0 | 959.6559 | 9.4 | -0.7 | MS-only | 81.8 | 959.66541:12245 960.66876:10610 961.67212:0 |
| Glycerophosphoinositols | PI 36:1 | 882.6066 | 7.8 | -0.6 | MS-only | 98.9 | 882.6087:6849 883.61205:6028 884.61541:0 |
| Glycerophosphoinositols | PI(132:0) | 793.5255 | 9.0 | -0.6 | MS-only | 94 | 793.53052:9940 794.53387:0 795.53723:0 |
| Glycerophosphoinositols | PI 37:1 | 896.6223 | 7.8 | -0.5 | MS-only | 99.4 | 896.62073:12848 897.62408:9529 898.62744:0 |
| Glycerophosphoinositols | PI 18:4 | 629.2333 | 4.2 | 1.0 | MS-only | 85.9 | 629.24146:30585 630.24481:12191 631.24817:0 |
| Glycerophosphoinositols | PI 48:4 | 1049.7029 | 11.1 | 1.0 | MS-only | 99.8 | 1049.70215:6682 1050.7055:7740 1051.70886:5873 |
| Glycerophosphoinositols | PI 38:6 | 905.5151 | 12.8 | 1.0 | MS-only | 93.5 | 905.50989:22586 906.51324:22204 907.5166:11974 |
| Glycerophosphoinositols | PI 13:0 | 567.2177 | 4.9 | 1.5 | MS-only | 99.5 | 567.21637:48146 568.21972:17506 569.22308:0 |
| Glycerophosphoinositols | PI 32:1 | 831.4994 | 14.2 | 2.7 | MS-only | 83.4 | 831.5083:8544 832.51165:6974 833.51501:0 |
| Glycerophosphoinositols | PI 23:0 | 698.3875 | 9.7 | 3.4 | MS-only | 99.8 | 702.41852:6362 703.42187:0 704.42523:0 |
| Glycerophospholipid | PA(34:0) | 677.5228 | 12.0 | -0.9 | MS-only | 99.1 | 677.52448:93875 678.52783:54443 679.53119:14835 |
| Glycerophospholipid | PA(42:4) | 763.5748 | 14.4 | -0.8 | MS-only | 99.6 | 763.57556:22412 764.57891:29692 765.58227:13827 |
| Glycerophospholipid | PA(44:2) | 813.6480 | 16.4 | -0.7 | MS-only | 99.6 | 813.6485:19491 814.65185:15174 815.65521:7628 |
| Glycerophospholipid | LysoPA(16:0/0:0) | 411.2618 | 7.1 | -0.6 | MS-only | 94.6 | 411.26657:157355 412.26992:49509 413.27328:25717 |
| Glycerophospholipid | PA(35:5) | 1041.7759 | 9.6 | -0.6 | MS-only | 100 | 1041.776:68316 1042.77935:67351 1043.78271:9980 |
| Glycerophospholipid | PA(39:1) | 727.5725 | 17.9 | 0.5 | MS-only | 99.6 | 727.57245:77002 728.5758:51486 729.57916:19405 |
| Glycerophosphoserines | PS 44:5 | 916.6038 | 13.9 | -5.3 | MS-only | 97.1 | 916.60046:4429 917.60381:0 918.60717:0 |
| Glycerophosphoserines | PS 20:4 | 582.2438 | 4.2 | -4.2 | MS-only | 99.2 | 582.24213:7757 583.24548:0 584.24884:0 |
| Glycerophosphoserines | PS 41:2 | 880.6038 | 9.2 | -3.2 | MS-only | 98.8 | 880.60162:7210 881.60497:8982 882.60833:0 |
| Glycerophosphoserines | PS 38:1 | 840.5725 | 13.2 | -2.7 | MS-only | 99.6 | 840.57159:5342 841.57494:0 842.5783:0 |
| Glycerophosphoserines | PS 38:2 | 838.5569 | 12.7 | -1.4 | MS-only | 95.2 | 838.55249:4078 839.55584:0 840.5592:0 |
| Glycerophosphoserines | PS 49:5 | 964.7001 | 8.7 | -1.1 | MS-only | 95.6 | 964.70441:11126 965.70776:6265 966.71112:0 |
| Glycerophosphoserines | PS 49:6 | 984.6664 | 7.8 | -0.8 | MS-only | 86.5 | 984.67432:8765 985.67767:5443 986.68103:0 |
| Glycerophosphoserines | PS 46:0 | 954.7134 | 9.4 | -0.6 | MS-only | 100 | 954.71332:426036 955.71667:352842 956.72003:56496 |
| Glycerophosphoserines | PS 19:4 | 546.2463 | 4.9 | -0.2 | MS-only | 86.6 | 546.25415:11030 547.2575:0 548.26086:0 |
| Glycerophosphoserines | PS 6:0 | 372.1054 | 8.8 | 1.4 | MS-only | 90.2 | 372.09888:3741 373.10223:0 374.10559:0 |
| Glycerophosphoserines | PS 28:5 | 692.3534 | 4.7 | 1.6 | MS-only | 96.3 | 692.34949:9204 693.35284:0 694.3562:0 |
| Glycerophosphoserines | Phosphatidylserine | 386.1211 | 5.6 | 3.0 | MS-only | 87.3 | 386.12869:30760 387.13204:9082 388.1354:0 |
| Hydroxycinnamic amides | Nb-Feruloyltryptamine | 337.1519 | 5.4 | -2.3 | MS/MS in silico prediction | 99.5 | 337.15195:3846730 338.1553:962012 339.15866:126175 |
| Hydroxycinnamic amides | Di-4-coumaroylputrescine | 381.1781 | 4.5 | -2.2 | MS/MS in silico prediction | 99.4 | 381.1777:1130864 382.18105:303878 383.18441:53696 |
| Hydroxycinnamic amides | N-(p-Hydroxyphenyl)ethyl p-hydroxycinnamide | 284.1293 | 1.6 | -1.1 | MS/MS in silico prediction | 99.1 | 284.12994:9523 285.13329:0 286.13665:0 |
| Monoacylglycerols | MAG 18:2 | 372.3075 | 9.0 | -1.8 | MS/MS confirmed | 99.6 | 372.30798:1668342 373.31133:491844 374.31469:80977 |
| Monoacylglycerols | MAG 15:1 | 332.2795 | 7.4 | -1.5 | MS-only | 98.2 | 332.27689:71329 333.28024:25789 334.2836:10616 |
| Monoacylglycerols | MAG 20:1 | 402.3578 | 6.7 | -1.0 | MS-only | 99.2 | 402.35956:26938 403.36291:5841 404.36627:0 |
| Monoacylglycerols | MAG 16:4 | 340.2482 | 7.7 | -0.6 | MS-only | 91.2 | 340.25443:347002 341.25778:64986 342.26114:42047 |
| Monoacylglycerols | MAG 26:0 | 488.4674 | 14.0 | 0.8 | MS-only | 84.7 | 488.47583:48624 489.47918:24005 490.48254:0 |
| Oxydized fatty acids | 6-Hydroxypentadecanedioic acid | 271.1891 | 18.4 | -1.0 | MS/MS in silico prediction | 100 | 271.18921:27140 272.19256:16141 273.19592:0 |
| Oxydized fatty acids | 13-OxoODE | 295.2263 | 17.0 | -0.9 | MS/MS in silico prediction | 99.8 | 295.22552:36626 296.22887:16110 297.23223:146238 |
| Phytoceramides | Cer(d18:1/20:0) | 576.4987 | 13.3 | -0.7 | MS-only | 97.7 | 576.50159:83049 577.50494:623344 578.5083:356897 |
| Phytoceramides | Cer(t18:0/23:0(2OH)) | 670.6365 | 22.1 | 0.7 | MS/MS in silico prediction | 97.8 | 670.63947:14409 671.64282:8577 672.64618:0 |
| Phytoceramides | CerP(d18:1/12:0) | 544.4935 | 12.8 | 1.0 | MS-only | 99.6 | 544.49457:9790 545.49792:5708 546.50128:0 |
| Ribonucleotides | 5-Aminoimidazole ribonucleotide | 296.0640 | 1.5 | 1.6 | MS/MS in silico prediction | 99.1 | 296.06372:5003362 297.06707:495572 298.07043:433517 |
| Sphingoid bases | Sphingosine 1-phosphate (d19:1-P) | 396.2257 | 5.2 | -5.0 | MS/MS in silico prediction | 99.4 | 396.22525:9414 397.2286:0 398.23196:0 |
| Sphingoid bases | Sphingosine 19:1 | 314.3054 | 7.7 | -4.1 | MS-only | 98.6 | 314.30304:89173 315.30639:24530 316.30975:0 |
| Sphingoid bases | Sphingosine 30:1 | 468.4775 | 11.7 | -3.0 | MS-only | 96.7 | 468.47385:17336 469.4772:0 470.48056:0 |
| Sphingoid bases | Sphinganine | 302.3054 | 8.2 | -2.3 | MS-only | 98.7 | 302.3031:165364 303.30645:44328 304.30981:14365 |
| Sphingoid bases | heptadecasphing-4-enine | 286.2741 | 7.1 | -1.5 | MS-only | 96.8 | 286.27039:7633 287.27374:0 288.2771:12458 |
| Sphingoid bases | Glucosylsphingosine | 444.3109 | 7.2 | 1.1 | MS-only | 99.5 | 444.30948:15834 445.31283:11655 446.31619:0 |
| Sphingoid bases | Sphingosine 24:1 | 384.3836 | 7.7 | 1.3 | MS-only | 100 | 384.38379:1006809 385.38714:293788 386.3905:39312 |
| Sphingoid bases | Dehydrophytosphingosine | 298.2711 | 16.1 | 1.4 | MS/MS in silico prediction | 100 | 298.27115:15697 299.2745:0 300.27786:0 |
| Triacylglycerols | TAG 43:4 | 746.6293 | 12.8 | -15.0 | MS-only | 99 | 746.63129:13000 747.63464:5642 748.638:0 |
| Triacylglycerols | TAG 68:19 | 1071.7412 | 4.1 | -6.6 | MS-only | 99.4 | 1071.74268:10477 1072.74603:8365 1073.74939:0 |
| Triacylglycerols | TAG 58:6 | 952.8328 | 18.3 | -5.0 | MS-only | 97.4 | 952.82959:22663900 953.83294:19718608 954.8363:8702713 |
| Triacylglycerols | TAG 74:18 | 1157.8507 | 9.8 | -4.2 | MS-only | 86.8 | 1157.85852:6870 1158.86187:7390 1159.86523:0 |
| Triacylglycerols | TG(16:0/18:0/18:1) | 878.8163 | 21.5 | -4.1 | MS/MS in silico prediction | 94.8 | 878.81158:4604832 879.81493:3181926 880.81829:1015528 |
| Triacylglycerols | TG(66:6) | 1063.8541 | 17.3 | -3.8 | MS-only | 99.6 | 1063.85522:5307 1064.85857:0 1065.86193:0 |
| Triacylglycerols | TAG 69:12 | 1094.9110 | 23.2 | -3.6 | MS-only | 92.1 | 1094.91675:27364 1095.9201:22611 1096.92346:10049 |
| Triacylglycerols | TAG 54:3 | 902.8164 | 20.5 | -2.8 | MS/MS confirmed | 96.3 | 902.8125:16307394 903.81585:11993437 904.81921:4457598 |
| Triacylglycerols | TG(16:0/20:1/20:4) | 909.7872 | 21.8 | -2.8 | MS/MS in silico prediction | 94.1 | 909.78217:927364 910.78552:642768 911.78888:214546 |
| Triacylglycerols | TAG 45:10 | 767.5221 | 9.9 | -2.7 | MS-only | 99.8 | 767.52118:3076 768.52453:0 769.52789:0 |
| Triacylglycerols | TG(52:6) | 833.7106 | 17.2 | -2.3 | MS-only | 90.3 | 833.7171:4456 834.72045:0 835.72381:0 |
| Triacylglycerols | TAG 69:14 | 1090.8798 | 17.3 | -2.0 | MS-only | 99.9 | 1090.87939:6891 1091.88274:6174 1092.8861:0 |
| Triacylglycerols | TG(21:0/22:1/22:2) | 1056.9871 | 26.3 | -2.0 | MS/MS in silico prediction | 93.2 | 1056.98169:510240 1057.98504:426869 1058.9884:160348 |
| Triacylglycerols | TAG 61:14 | 978.7545 | 16.8 | -1.9 | MS-only | 90.1 | 978.76111:17901 979.76446:13099 980.76782:0 |
| Triacylglycerols | TAG 38:3 | 683.5221 | 13.9 | -1.8 | MS-only | 97.8 | 683.51923:32442 684.52258:21528 685.52594:9287 |
| Triacylglycerols | TG(45:5) | 755.6038 | 15.8 | -1.6 | MS-only | 98.4 | 755.60144:4864 756.60479:6126 757.60815:5219 |
| Triacylglycerols | TAG 63:11 | 1012.8328 | 18.3 | -1.5 | MS-only | 99.9 | 1012.83301:3910 1013.83636:0 1014.83972:0 |
| Triacylglycerols | TAG 59:1 | 976.9267 | 24.7 | -1.4 | MS-only | 87.8 | 976.91937:90392 977.92272:66181 978.92608:27773 |
| Triacylglycerols | TG(66:14) | 1031.7915 | 16.5 | -1.4 | MS-only | 99.8 | 1031.79126:8792 1032.79461:0 1033.79797:0 |
| Triacylglycerols | TAG 44:1 | 766.6919 | 16.6 | -1.0 | MS-only | 98.7 | 766.69409:69956 767.69744:36944 768.7008:595040 |
| Triacylglycerols | TAG 62:5 | 1015.8664 | 17.1 | -0.8 | MS-only | 99.9 | 1015.86627:11253 1016.86962:12228 1017.87298:7827 |
| Triacylglycerols | TAG 64:3 | 1042.9736 | 25.3 | -0.8 | MS-only | 87.8 | 1042.96631:4255284 1043.96966:3210304 1044.97302:1262052 |
| Triacylglycerols | TAG 64:17 | 1014.7545 | 9.5 | -0.7 | MS-only | 99.2 | 1014.75629:8430 1015.75964:0 1016.763:0 |
| Triacylglycerols | TG(60:4) | 967.8566 | 14.1 | -0.4 | MS-only | 100 | 967.85657:53212 968.85992:38533 969.86328:12326 |
| Triacylglycerols | TAG 64:1 | 1047.0049 | 16.2 | -0.2 | MS-only | 87.5 | 1046.99731:100762 1048.00066:82154 1049.00402:33165 |
| Triacylglycerols | TAG 54:1 | 911.8038 | 21.6 | -0.1 | MS-only | 94.3 | 911.79901:43597 912.80236:35414 913.80572:11274 |
| Triacylglycerols | TAG 61:9 | 988.8328 | 17.1 | 0.2 | MS-only | 99.3 | 988.83124:39816 989.83459:52839 990.83795:21849 |
| Triacylglycerols | TAG 55:0 | 927.8351 | 16.8 | 0.3 | MS-only | 94.7 | 927.83978:39024 928.84313:51429 929.84649:27447 |
| Triacylglycerols | TG(51:6) | 819.6950 | 12.9 | 0.4 | MS-only | 98.6 | 819.69269:63143 820.69604:54852 821.6994:17113 |
| Triacylglycerols | TAG 49:3 | 837.6943 | 16.1 | 0.5 | MS-only | 99.8 | 837.69373:10151 838.69708:16336 839.70044:10248 |
| Triacylglycerols | TG(64:5) | 1021.9035 | 19.0 | 0.6 | MS-only | 99.8 | 1021.90308:23879 1022.90643:18911 1023.90979:7591 |
| Triacylglycerols | TAG 35:1 | 640.5511 | 11.5 | 0.6 | MS-only | 94.7 | 640.54639:22183 641.54974:12413 642.5531:37842 |
| Triacylglycerols | TG(57:8) | 917.7446 | 11.8 | 0.6 | MS-only | 98.7 | 917.74237:11895 918.74572:7264 919.74908:0 |
| Triacylglycerols | TAG 42:0 | 740.6763 | 19.1 | 0.6 | MS-only | 99.7 | 740.67633:18153 741.67968:10968 742.68304:5632 |
| Triacylglycerols | TG(13:0/13:0/14:0) | 712.6545 | 15.4 | 0.6 | MS/MS in silico prediction | 99.9 | 712.65411:68728 713.65746:45256 714.66082:16942 |
| Triacylglycerols | TAG 54:0 | 908.8641 | 22.2 | 0.7 | MS-only | 92.7 | 908.85858:726820 909.86193:517355 910.86529:158795 |
| Triacylglycerols | TG(63:5) | 1007.8854 | 17.2 | 0.7 | MS-only | 99.9 | 1007.88538:14903 1008.88873:12209 1009.89209:0 |
| Triacylglycerols | TAG 31:0 | 591.4595 | 11.1 | 0.7 | MS-only | 96.9 | 591.45599:464498 592.45934:236678 593.4627:75551 |
| Triacylglycerols | TAG 55:1 | 920.8641 | 21.6 | 0.8 | MS-only | 84.7 | 920.85559:518784 921.85894:357210 922.8623:118235 |
| Triacylglycerols | TAG 44:2 | 769.6317 | 19.4 | 0.8 | MS-only | 97.4 | 769.6286:9671 770.63195:0 771.63531:0 |
| Triacylglycerols | TAG 58:3 | 958.8797 | 21.2 | 0.8 | MS-only | 95.4 | 958.87543:14115958 959.87878:10903180 960.88214:4058028 |
| Triacylglycerols | TAG 46:2 | 792.7076 | 16.8 | 0.8 | MS-only | 99.8 | 792.70709:54724 793.71044:32379 794.7138:13633 |
| Triacylglycerols | TG(61:5) | 979.8566 | 27.5 | 0.8 | MS-only | 99.3 | 979.85675:21959 980.8601:15486 981.86346:0 |
| Triacylglycerols | TAG 37:1 | 673.5378 | 13.5 | 0.9 | MS-only | 98.5 | 673.5354:76950 674.53875:52520 675.54211:18105 |
| Triacylglycerols | TAG 54:1 | 906.8485 | 20.9 | 0.9 | MS-only | 95.2 | 906.84406:8413111 907.84741:6004089 908.85077:2020894 |
| Triacylglycerols | TAG 71:12 | 1122.9423 | 23.0 | 0.9 | MS-only | 98.2 | 1122.9447:12475 1123.94805:0 1124.95141:0 |
| Triacylglycerols | TAG 58:2 | 960.8954 | 22.5 | 0.9 | MS-only | 92.7 | 960.88989:7413547 961.89324:5344776 962.8966:1877045 |
| Triacylglycerols | TAG 44:7 | 759.5534 | 20.5 | 1.0 | MS-only | 99 | 759.55481:7984 760.55816:6249 761.56152:0 |
| Triacylglycerols | TAG 64:2 | 1044.9893 | 26.9 | 1.0 | MS-only | 89.7 | 1044.98267:1918218 1045.98602:1491062 1046.98938:560523 |
| Triacylglycerols | TG(66:10) | 1021.8671 | 17.5 | 1.0 | MS-only | 83.6 | 1021.87598:4039 1022.87933:0 1023.88269:0 |
| Triacylglycerols | TAG 64:9 | 1035.8351 | 17.3 | 1.0 | MS-only | 98 | 1035.83228:11455 1036.83563:8945 1037.83899:0 |
| Triacylglycerols | TAG 64:5 | 1043.8977 | 19.8 | 1.0 | MS-only | 84.6 | 1043.90625:6815 1044.9096:8894 1045.91296:5618 |
| Triacylglycerols | TAG 66:3 | 1071.0049 | 16.2 | 1.1 | MS-only | 83.6 | 1070.99597:366415 1071.99932:285640 1073.00268:105872 |
| Triacylglycerols | TAG 72:16 | 1128.8954 | 17.8 | 1.1 | MS-only | 98.9 | 1128.89331:8471 1129.89666:7730 1130.90002:0 |
| Triacylglycerols | TAG 35:0 | 647.5221 | 13.5 | 1.1 | MS-only | 99.2 | 647.52045:57038 648.5238:0 649.52716:0 |
| Triacylglycerols | TAG 53:1 | 892.8328 | 20.9 | 1.1 | MS-only | 95.4 | 892.82849:753814 893.83184:517959 894.8352:176824 |
| Triacylglycerols | TG(44:3) | 1019.8879 | 16.2 | 1.1 | MS-only | 99.9 | 1019.888:83820 1020.89135:62682 1021.89471:23041 |
| Triacylglycerols | TAG 58:4 | 956.8589 | 23.2 | 1.1 | MS/MS confirmed | 100 | 956.85876:632183 957.86211:491871 958.86547:171175 |
| Triacylglycerols | TAG 57:2 | 946.8797 | 22.5 | 1.1 | MS-only | 92.7 | 946.87421:801773 947.87756:579297 948.88092:221817 |
| Triacylglycerols | TAG 41:7 | 717.5065 | 13.9 | 1.2 | MS-only | 97.7 | 717.50348:52509 718.50683:28684 719.51019:14065 |
| Triacylglycerols | TG(18:0/18:1/18:1) | 904.8320 | 22.4 | 1.2 | MS/MS in silico prediction | 96.4 | 904.82812:8989583 905.83148:6118930 906.83484:2115903 |
| Triacylglycerols | TAG 71:6 | 1135.0363 | 21.6 | 1.3 | MS-only | 84.8 | 1135.04468:9209 1136.04803:0 1137.05139:0 |
| Triacylglycerols | TAG 39:6 | 691.4908 | 13.7 | 1.3 | MS-only | 96.8 | 691.48724:122395 692.49059:69207 693.49395:31254 |
| Triacylglycerols | TG(47:2) | 789.6844 | 16.0 | 1.4 | MS-only | 97.5 | 789.68134:23321 790.68469:18297 791.68805:6935 |
| Triacylglycerols | TAG 60:2 | 988.9267 | 24.2 | 1.4 | MS-only | 92.3 | 988.92108:4319112 989.92443:3233174 990.92779:1151011 |
| Triacylglycerols | TAG 47:4 | 802.6919 | 15.0 | 1.4 | MS-only | 99.7 | 802.69092:13916 803.69427:8756 804.69763:0 |
| Triacylglycerols | TAG 59:5 | 968.8641 | 13.7 | 1.4 | MS-only | 99.7 | 968.86292:38888 969.86627:16958 970.86963:0 |
| Triacylglycerols | TAG 61:8 | 990.8485 | 24.7 | 1.5 | MS-only | 99.2 | 990.84723:10594 991.85058:0 992.85394:0 |
| Triacylglycerols | TAG 66:4 | 1068.9893 | 27.4 | 1.5 | MS-only | 91.5 | 1068.9834:532749 1069.98675:419356 1070.99011:161004 |
| Triacylglycerols | TG(62:7) | 1017.8722 | 27.4 | 1.5 | MS-only | 99.3 | 1017.87231:118282 1018.87566:85733 1019.87902:30302 |
| Triacylglycerols | TG(47:6) | 763.6324 | 12.8 | 1.6 | MS-only | 99.7 | 763.63318:6653 764.63653:0 765.63989:6043 |
| Triacylglycerols | TG(12:0/16:0/16:0) | 768.7169 | 17.9 | 1.6 | MS/MS in silico prediction | 100 | 768.7171:215177 769.72045:132877 770.72381:43589 |
| Triacylglycerols | TG(15:0/18:1/18:3) | 858.7574 | 18.0 | 1.7 | MS/MS in silico prediction | 99.9 | 858.758:14773 859.76135:11976 860.76471:6248 |
| Triacylglycerols | TAG 61:1 | 1004.9580 | 27.3 | 1.7 | MS-only | 90.1 | 1004.95154:84687 1005.95489:62120 1006.95825:24471 |
| Triacylglycerols | TG(21:0/22:2/22:2) | 1054.9670 | 26.1 | 1.7 | MS/MS in silico prediction | 99.9 | 1054.96729:302338 1055.97064:242274 1056.974:93378 |
| Triacylglycerols | TG(16:1/18:1/19:0) | 890.8214 | 18.5 | 1.8 | MS/MS in silico prediction | 99.4 | 890.823:16697 891.82635:0 892.82971:0 |
| Triacylglycerols | TAG 55:3 | 916.8328 | 18.5 | 1.8 | MS-only | 94.1 | 916.83771:27165 917.84106:22940 918.84442:13524 |
| Triacylglycerols | TG(20:3/22:0/22:4) | 1017.8860 | 18.3 | 1.8 | MS/MS in silico prediction | 93.8 | 1017.88086:26686 1018.88421:17004 1019.88757:8140 |
| Triacylglycerols | TAG 46:5 | 791.6160 | 15.4 | 1.8 | MS-only | 97.2 | 791.61932:5350 792.62267:0 793.62603:0 |
| Triacylglycerols | TAG 46:9 | 778.5980 | 15.2 | 1.9 | MS-only | 99.6 | 778.59705:8295 779.6004:7230 780.60376:0 |
| Triacylglycerols | TAG 56:1 | 934.8797 | 22.3 | 1.9 | MS-only | 92.7 | 934.87421:5468854 935.87756:3766913 936.88092:1297918 |
| Triacylglycerols | TG(47:6) | 781.6194 | 13.0 | 1.9 | MS-only | 81.7 | 781.60986:41306 782.61321:19279 783.61657:5471 |
| Triacylglycerols | TAG 26:0 | 516.4258 | 8.4 | 1.9 | MS-only | 92.8 | 516.4314:6508 517.43475:0 518.43811:0 |
| Triacylglycerols | TAG 74:7 | 1180.0230 | 24.1 | 2.0 | MS-only | 99.6 | 1180.02319:30851 1181.02654:15582 1182.0299:5778 |
| Triacylglycerols | TAG 57:9 | 937.7256 | 17.7 | 2.0 | MS-only | 99 | 937.72748:59843 938.73083:41098 939.73419:19692 |
| Triacylglycerols | TAG 55:11 | 905.6630 | 16.3 | 2.1 | MS-only | 97.5 | 905.65985:4307 906.6632:5544 907.66656:0 |
| Triacylglycerols | TAG 66:1 | 1075.0363 | 22.6 | 2.3 | MS-only | 87.6 | 1075.02881:14630 1076.03216:12418 1077.03552:5848 |
| Triacylglycerols | TG(52:2) | 859.7626 | 18.0 | 2.4 | MS-only | 99.3 | 859.76117:11085 860.76452:7054 861.76788:0 |
| Triacylglycerols | TAG 30:0 | 572.4885 | 12.1 | 2.4 | MS-only | 97 | 572.49194:13738 573.49529:9954 574.49865:0 |
| Triacylglycerols | TAG 46:7 | 782.6293 | 16.1 | 2.5 | MS-only | 92.1 | 782.62354:8221 783.62689:6304 784.63025:0 |
| Triacylglycerols | TG(15:0/18:1/18:2) | 860.7688 | 18.5 | 2.6 | MS/MS in silico prediction | 99.4 | 860.77032:8002 861.77367:6074 862.77703:7841 |
| Triacylglycerols | TAG 42:1 | 743.6160 | 16.4 | 3.2 | MS-only | 96.8 | 743.61957:3893 744.62292:0 745.62628:0 |
| Triacylglycerols | TAG 34:3 | 622.5042 | 17.2 | 3.3 | MS-only | 94.2 | 622.49933:11349 623.50268:5865 624.50604:0 |
| Triacylglycerols | TAG 74:6; | 1182.0386 | 25.6 | 3.8 | MS-only | 99.1 | 1182.03723:12874 1183.04058:8438 1184.04394:0 |
| Triacylglycerols | TAG 72:5 | 1156.0230 | 25.7 | 3.8 | MS-only | 98.3 | 1156.02075:13769 1157.0241:7570 1158.02746:0 |
| Triacylglycerols | TAG 74:3 | 1188.0855 | 27.4 | 4.6 | MS-only | 94.8 | 1188.08105:12232 1189.0844:5583 1190.08776:0 |
| Triterpenoids | 26-Methyl nigranoate | 485.3621 | 16.4 | -2.4 | MS/MS in silico prediction | 98.4 | 485.35953:6107 486.36288:8308 487.36624:5660 |
| Triterpenoids | Tsugaric acid C | 497.3573 | 7.9 | 0.7 | MS/MS in silico prediction | 98.3 | 497.35498:877497 498.35833:397021 499.36169:74108 |
| Triterpenoids | Violaxanthin | 601.4246 | 15.6 | 0.7 | MS/MS in silico prediction | 96.6 | 601.4209:37289 602.42425:14238 603.42761:6122 |
| Triterpenoids | beta-carotenone | 563.4521 | 8.9 | 0.9 | MS/MS in silico prediction | 99.7 | 563.45245:1069879 564.4558:603554 565.45916:127492 |
| Triterpenoids | Faradiol-laurate | 607.5447 | 17.9 | 1.8 | MS/MS in silico prediction | 90.2 | 607.53809:327314 608.54144:167218 609.5448:38686 |

**Supplementary Material Figure 1S:** Principal-components-analysis (PCA) score plot built with filtered data and colored according to a) geographical area (LM: Lombardia; PM: Piedmont), and b) harvest year.
